# Immunity and clinical efficacy of an inactivated enterovirus 71 vaccine in healthy Chinese children: a report of further observations

**DOI:** 10.1186/s12916-015-0448-7

**Published:** 2015-09-17

**Authors:** Longding Liu, Zhaojun Mo, Zhenglun Liang, Ying Zhang, Rongcheng Li, Kien Chai Ong, Kum Thong Wong, Erxia Yang, Yanchun Che, Jingjing Wang, Chenghong Dong, Min Feng, Jing Pu, Lichun Wang, Yun Liao, Li Jiang, Soon Hao Tan, Perera David, Teng Huang, Zhenxin Zhou, Xuanyi Wang, Jielai Xia, Lei Guo, Ling Wang, Zhongping Xie, Wei Cui, Qunying Mao, Yan Liang, Hongling Zhao, Ruixiong Na, Pingfang Cui, Haijing Shi, Junzhi Wang, Qihan Li

**Affiliations:** Yunnan Key Laboratory of Vaccine Research and Development on Severe Infectious Diseases, Institute of Medical Biology, Chinese Academy of Medical Sciences and Peking Union Medical College, Kunming, China; Guangxi Province Centres for Disease Control and Prevention, Nanning, China; National Institutes for Food and Drug Control, Beijing, China; Departments of Biomedical Science and Pathology, Faculty of Medicine, University of Malaya, Kuala Lumpur, Malaysia; Jiangsu Convac Biotechnology Co., Ltd, Taizhou, Jiangsu China; Institute of Health & Community Medicine, University Malaysia Sarawak, Kuala Lumpur, Malaysia; Key Laboratory of Medical Molecular Virology, Ministries of Education and Health, Institute of Biological Sciences, Shanghai Medical College, Fudan University, Shanghai, China; Department of Health Statistics, Fourth Military Medical University, Xi’an, China

**Keywords:** Cross-neutralization, Enterovirus 71, Hand, foot, and mouth disease, Inactivated vaccine, Long-term effect

## Abstract

**Background:**

To investigate the long-term effects on immunity of an inactivated enterovirus 71 (EV71) vaccine and its protective efficacy.

**Methods:**

A sub-cohort of 1,100 volunteers from Guangxi Province in China was eligible for enrolment and randomly administered either the EV71 vaccine or a placebo on days 0 and 28 in a phase III clinical trial and then observed for the following 2 years with approval by an independent ethics committee of Guangxi Zhuang Autonomous Region, China. Serum samples from the 350 participants who provided a full series of blood samples (at all the sampling points) within the 2-year period were collected. Vaccine-induced immune effects, including the neutralizing antibody titres and cross-protection against different genotypes of EV71, were examined. This study also evaluated the protective efficacy of this vaccine based upon clinical diagnosis.

**Results:**

This sub-cohort showed a >60 % drop-out rate over 2 years. The seroconversion rates among the 161 immunized subjects remained >95 % at the end of study. The geometric mean titres of neutralizing antibodies (anti-genotype C4) 360 days after vaccination in 350 subjects were 81.0 (subjects aged 6–11 months), 98.4 (12–23 months), 95.0 (24–35 months), and 81.8 (36–71 months). These titres subsequently increased to 423.1, 659.0, 545.0, and 321.9, respectively, at 540 days post-immunization (d.p.i.), and similar levels were maintained at 720 d.p.i. Higher IFN-γ/IL-4-specific responses to the C4 genotype of EV71 and cross-neutralization reactivity against major EV71 genotype strains were observed in the vaccine group compared to those in the placebo group. Five EV71-infected subjects were observed in the placebo-treated control group and none in the vaccine-immunized group in per-protocol analysis.

**Conclusion:**

These results are consistent with the induction of dynamic immune responses and protective efficacy of the vaccine against most circulating EV71 strains.

**Trial registration number:**

Clinicaltrials.gov, NCT01569581, Trial registration date: March 2012

**Electronic supplementary material:**

The online version of this article (doi:10.1186/s12916-015-0448-7) contains supplementary material, which is available to authorized users.

## Background

Hand, foot, and mouth disease (HFMD) has recently emerged in the Asian-Pacific region as the most severe epidemic disease affecting children [1, 2]. The effective prevention and control of HFMD epidemics is widely recognized as an essential public health issue [3, 4]. Enterovirus 71 (EV71) and coxsackievirus A16 (CA16) are the two major pathogens causing HFMD. Of these two viruses, EV71 infection is associated with a higher death rate and is primarily responsible for fatalities [5–7]. Attempts to develop inactivated EV71 vaccines have made significant progress over a short period [8–10]. In a published report of a 1-year phase III clinical trial, an inactivated EV71 vaccine derived from a C4 genotype strain (the predominant strain circulating on the Chinese mainland) displayed adequate safety, immunogenicity, and efficacy, showing an efficacy of 97.4 % and geometric mean titres (GMTs) of 224.4 and 118.0 at 56 and 180 days post-immunization (d.p.i.), respectively [8]. However, the long-term effects of a vaccine on immunity (specifically, its cross-protection against circulating strains of various genotypes) are generally considered critical characteristics for its licensing and clinical application.

Herein, we report the results of a continued evaluation of the immunogenicity, immune memory effects, and efficacy of this EV71 vaccine in a sub-cohort of subjects. Initially, 1,100 subjects were selected for the random administration of either the vaccine or a placebo, and 350 subjects provided a complete series of blood samples within the 2-year study period. In the present study, we performed a cross-neutralization assay with nine individual EV71 strains of genotypes A, B (B3–B5), and C (C1–C5), using 160 serum samples obtained from the 350 subjects from the immunized and placebo groups, in a 1:1 ratio. The results show the efficacy of the immune response induced by the inactivated EV71 vaccine and provide substantial data on the potential utility of this vaccine.

## Methods

### Vaccine and vaccination

The inactivated EV71 vaccine was developed in a good manufacturing practice-compliant facility at the Institute of Medical Biology, Chinese Academy of Medical Sciences, and was tested at the National Institutes for Food and Drug Control before this study. The vaccine was prepared from the EV71 FY-23K-B strain of sub-genotype C4, cultured in a human diploid cell line (KMB17) for proliferation, and then purified and inactivated. Each dose of the vaccine contained 100 U of inactivated EV71 viral antigen adsorbed onto 0.5 mg of aluminum hydroxide, suspended in 0.5 mL of buffered saline. Both the vaccine and placebo were administered intramuscularly at 0 and 28 days.

### Subjects and study design

The primary outcome of this trial was the determination of the efficacy of an inactivated EV71 vaccine, and the secondary outcome was the evaluation of the long-term immune persistence of this vaccine over a 2-year observation period. The study was conceived and performed with a randomized, double-blind, placebo-controlled cohort and registered at Clinicaltrials.gov as NCT01569581 [8]. The study was proposed by the Centres for Disease Control and Prevention of Guangxi Province in association with a professional statistics group from the Fourth Military Medical University, and was approved by the China Food and Drug Administration and an independent ethics committee of Guangxi Zhuang Autonomous Region, China (Additional file 1). A subset of 1,100 children, whose legal guardian provided written informed consent (Additional file 1), from the 12,000 subjects of the phase III trial undertaken in the Guilin region of Guangxi Province, China, were eligible for enrolment and randomly selected based on the inclusion criteria for participation (see Methods in the Additional file 1). The subjects were grouped according to age: 6–11 months (n = 350), 12–23 months (n = 350), 24–35 months (n = 300), and 36–71 months (n = 100). The subjects within each age group were randomly assigned to receive either the vaccine or the placebo, in a ratio of 1:1. To measure the immune responses, blood samples were collected from each available subject at baseline and at 56, 180, 360, 540, and 720 d.p.i., administered between March and April 2012. A suspected case of HFMD was defined as having a febrile illness (>37.5 °C) accompanied by a papular/vesicular rash with the characteristic distribution on the oral mucosa and limb extremities (see Methods in the Additional file 1). A case of EV71 HFMD was defined as a suspected case of HFMD in whom EV71 was detected with a throat swab or stool specimen, using quantitative reverse transcription–polymerase chain reaction (qRT–PCR). A case of severe EV71 HFMD was defined as a suspected case of HFMD with neurological, respiratory, or circulatory complications based on the HFMD diagnostic criteria of the Ministry of Health of China [11]. To integrate the identification of active cases across the region, a healthcare center-based surveillance system was established in each county to detect suspected cases of HFMD. SimoonRecord, an independent contract research organization, assessed all clinical data at the end of each year. The surveillance method and the processing of suspected cases have been described elsewhere [8]. Because the vaccine is not yet licensed, the trial cohort was monitored under the same disease surveillance system after unblinding.

### Measurement of the persistent immune response

Serum samples from the 350 participants who provided a full series of blood samples (at all the sampling points) within the 2-year period were collected. These 350 subjects were investigated for the 2-year study according to per-protocol analysis. The samples were assayed for EV71-specific neutralizing antibodies with a micro-neutralization assay on Vero cells grown in 96-well plates and a standard viral strain (C4) provided by the National Institutes for Food and Drug Control [8]. Susceptible participants, who showed a level of neutralizing antibodies against the C4 genotype of EV71 of <1:8 (the threshold of detection) before immunization, were assigned a value of 1:4.

Overall, 160 individuals (40 individuals in each age group: 20 individuals in the vaccine group and 20 in the placebo group, all of whom provided a full series of blood samples within the 2-year study period) were randomly selected using the RAND function in the EXCEL software (Microsoft, Redmond, WA, USA). Their serum samples were collected at 0, 56, and 360 d.p.i. for the cross-neutralization assay. Nine strains of the EV71 virus (genotypes A, B3, B4, B5, C1, C2, C3, C4, and C5) were used in this assay (Additional file 1). Of these strains, all but genotype A were isolated from epidemics occurring in various areas within the Asian-Pacific region [12–15]. The viral strains, grown on Vero cell monolayers, were harvested after the typical cytopathic effect was observed, and the titres were determined. Three hundred cell culture infectious doses of all the viruses were used for the neutralization test.

A total of 40 blood samples (10 samples from each age group: five samples from the vaccine group and five samples from the placebo group) were selected randomly from the 350 subjects who had provided a complete series of blood samples within the 2-year study period. The serum samples collected at 720 d.p.i. were used in interferon-γ (IFN-γ) and interleukin-4 (IL-4) enzyme-linked immunospot (ELISPOT) assays (Mabtech AB, Stockholm, Sweden). The ELISPOT assays were performed as previously described. Briefly, a 96-well polyvinylidene-difluoride-backed plate was pre-coated with anti-IFN-γ or anti-IL-4 monoclonal antibody, incubated overnight, and blocked for 1 h at 37 °C. The wells containing a predetermined density of peripheral blood mononuclear cells (PBMCs) and a stimulatory peptide (10 mg/mL; amino acid sequence: STAETTLDSFF) [16] were incubated at 37 °C for another 24 h. The cells were then removed and the colors were developed according to the manufacturer’s instructions. The colored spots were counted with an automated ELISPOT reader (Cellular Technology Limited, OH, USA). The spot-forming cells were EV71 epitope-specific IFN-γ-producing T cells.

### Statistical analysis

The analysis of this observational study was begun in March 2012, and the follow-up by professional statisticians from the Fourth Military Medical University is ongoing. Based on the fact that a 0.7 % HFMD morbidity was observed in children aged 6–72 months in Guangxi Province, China, in 2009–2011 [8, 17, 18], and that 55–72 % of HFMD cases were identified as being infected by EV71 [8, 17, 18], 6,000 subjects per arm were required to detect any difference in vaccine efficacy between the vaccine and placebo groups with a statistical power of 90 % and a two-tailed alpha of 0.05. Because a 20 % dropout rate was considered probable within the 12 months after vaccination in the phase III trial and at least 2 years were required to obtain adequate data from which to estimate the long-term efficacy of the seroprotection induced by the EV71 vaccine, we specified 1,100 children as the recruitment target for this study. This sample size represents approximately 10 % of the 12,000 total subjects in the phase III trial, and comprised 550 subjects per arm, in a ratio of 1:1 for the vaccine- and placebo-treated groups. A difference was detected in the neutralization antibodies induced in the vaccine and placebo groups, with a statistical power of 90 % and a two-tailed alpha of 0.05. The Kaplan–Meier method was used to compare the vaccine efficacy, and the Mantel–Haenszel χ^2^ method was used to assess the differences in the two groups. Student’s *t*-test or the Mann–Whitney *U* test (when the data were not normally distributed) was used to assess the dimensional outcomes, and the χ^2^ test or Fisher’s exact test (when the data were sparse) was used to assess the dichotomous outcomes. Both types of outcomes were evaluated in a two-tailed manner. In the statistical analysis of the antibody titres, the values were converted to logarithms to facilitate the assessment of the GMTs. The SAS software version 9.1 (SAS Institute) was used for all statistical analyses.

## Results and Discussion

Clinical phase III trials have shown that inactivated EV71 vaccines are effective in the control and prevention of HFMD caused by EV71 infection in child populations [8–10]. The present study was based on four individual age groups in a sub-cohort comprising 1,100 subjects who had been included in a previous clinical trial [8]. The major vaccinated cohorts in these groups were at a susceptible age for the development of HFMD, according to epidemiological studies [19], because understanding the dynamic profiles of anti-EV71 antibodies in children vaccinated during this period of life is essential to prevent disease development.

**Fig. 1:**
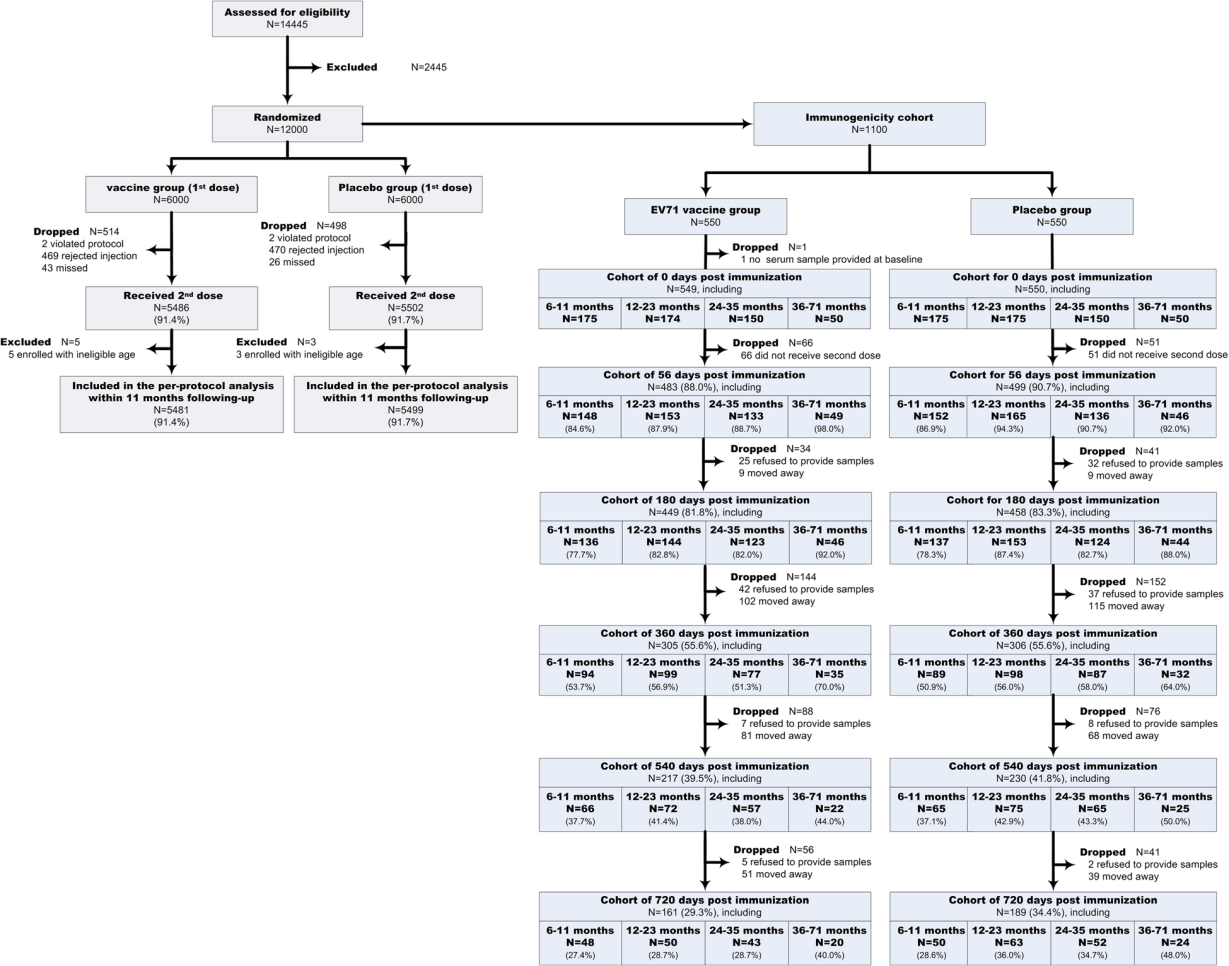
Schematic representation of the phase III clinical trial of an inactivated enterovirus 71 vaccine. In total, 14,445 children, aged 6–71 months, were assessed for a phase III clinical trial and 12,000 eligible volunteers were randomly assigned to receive the vaccine or a placebo [8]. The blood samples of 1,100 individuals were collected at 0, 56, 180, 360, 540, and 720 days after immunization to evaluate the production of anti-EV71 neutralizing antibodies. The follow-up rates (%) are indicated for each time point

In the present study, 1,100 participants in the four age groups were allocated to either the experimental or control groups, in a 1:1 ratio (Additional file 1: Table S1). According to the primary design, the phase III trial was initiated in March 2012 and ended after a 1-year follow-up period. However, a long-term experiment using this sub-cohort was continued for 2 years. Throughout the 720-day study period, the overall drop-out rates, for any reason, were 72.0 %, 67.6 %, 68.3 %, and 56.0 % in the 6–11 month, 12–23 month, 24–35 month, and 36–71 month age groups, respectively (Fig. 1). The high drop-out rates, which included some individuals who moved away and some who refused to provide samples (Fig. 1), predominantly reflected the relocation of families, which has been occurring more frequently in rural and urban areas in recent years. After drop-out, 350 subjects remained in this sub-cohort (161 participants in the immunized group and 189 in the placebo group), and participated in an immunological study at all 56, 180, 360, 540 and 720 d.p.i. time points according to per-protocol analysis (Fig. 1).

**Fig. 2:**
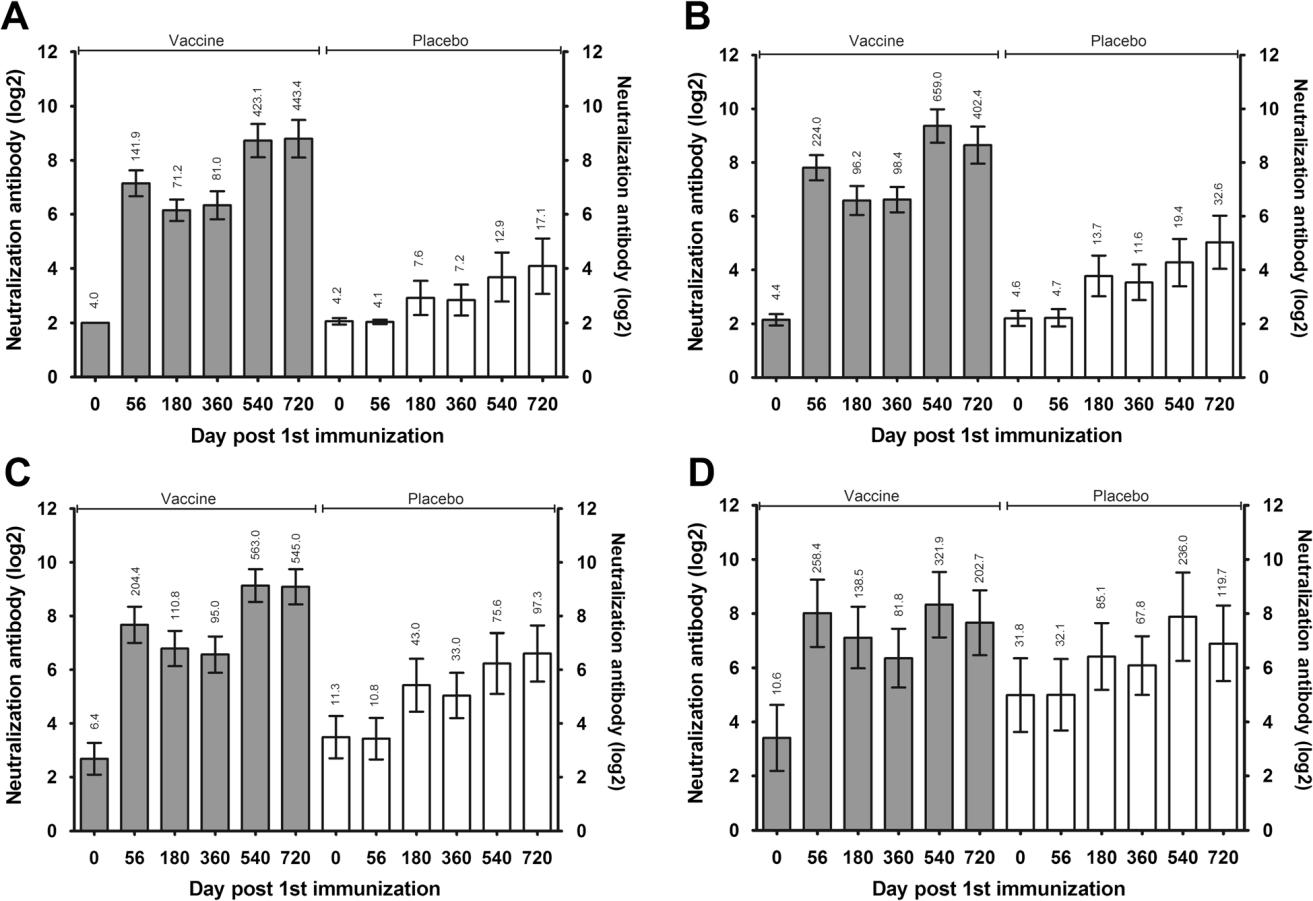
Dynamic profiles of the antibody levels during a 2-year follow-up period in 350 children immunized with the vaccine. Blood samples from 350 participants, who provide a full series of blood samples within the 2-year follow-up period, were collected to evaluate the production of anti-EV71 neutralizing antibodies. These children were 6–11 months old (**a**) (48 individuals in the vaccine group and 50 in the placebo group), 12–23 months old (**b**) (50 individuals in the vaccine group and 63 in the placebo group), 24–35 months old (**c**) (43 individuals in the vaccine group and 52 in the placebo group), and 36–71 months old (**d**) (20 individuals in the vaccine group and 24 in the placebo group). The geometric mean titres are shown above the bar. The bar indicates the 95 % confidence interval

The levels of neutralizing antibodies (against genotype C4 virus) in the sera of the vaccinated subjects 56 d.p.i. were 141.9 (95 % confidence interval [CI], 102.0–197.3) in the 6–11 months group, 224.0 (95 % CI, 162.1–309.6) in the 12–23 month age group, 204.4 (95 % CI, 128.9–324.2) in the 24–35 month age group, and 258.4 (95 % CI, 109.4–610.3) in the 36–71 month age group, whereas those in the sera of the placebo control subjects were 4.1 (95 % CI, 3.9–4.3 ), 4.7 (95 % CI, 3.7–5.8 ), 10.8 (95 % CI, 6.3–18.5), and 32.1 (95 % CI, 12.8–80.5), respectively (Fig. 2). These clear differences suggest the effective immunogenicity of the vaccine. However, the antibody titres of the vaccinated children subsequently decreased to 71.2 (95 % CI, 54.2–93.5), 96.2 (95 % CI, 66.2–139.8), 110.8 (95 % CI, 70.6–173.8), and 138.5 (95 % CI, 63.0–304.1), respectively, at 180 d.p.i., and similar levels were observed at 360 d.p.i. (Fig. 2). This probably reflects the normal dynamic decay of the immune responses induced by viral antigens [20–22]. Interestingly, the antibody levels increased to 423.1 (95 % CI, 276.2–648.1), 659.0 (95 % CI, 426.9–1017.0), 563.0 (95 % CI, 368.2–860.8), and 321.9 (95 % CI, 139.5–742.7), respectively, at 540 d.p.i. and to 443.4 (95 % CI, 274.2–717.0), 402.4 (95 % CI, 249.5– 648.9), 545.0 (95 % CI, 344.7–861.4), and 202.7 (95 % CI, 88.4–465.0), respectively, at 720 d.p.i. The antibody titres of the placebo control subjects also increased in the older age groups (Fig. 2c,d), but with only slight variations observed in younger age groups (Fig. 2a,b). The GMTs of the placebo subjects were 97.3 in the 24–35 month age group and 119.70 in the 36–71 month age group at 720 d.p.i., considerably higher than those in the 6–11 (17.07) and 12–23 (32.63) month age groups (Fig. 2). These results suggest that older children more easily contract the circulating virus than younger ones [23] (Fig. 2). Strikingly, the serum conversion rates in the children of all age groups observed at different time points were typically greater than 95 % in the immunized cohort (Table 1). These data suggest that higher serum antibody conversion rates and neutralizing antibody GMTs with protective significance were maintained in the cohort immunized with the vaccine 2 years earlier. This demonstrates the efficacy of this vaccine and the enhanced effect of the antibody response in immunized individuals, which might have been induced by naturally circulating virus, as previously described [24–28].

**Table 1:** Serum conversion rates in children immunized with the EV71 vaccine who provided a full series of blood samples within the 2-year follow-up period^a^

Age group (Months)	Days post immunization	Vaccine^b^	Placebo^b^	*P* value
6–11	0	0/48 (0.0)	1/50 (2.0)	1.000
	56	48/48 (100.0)	1/50 (2.0)	<0.0001
	180	47/48 (97.9)	8/50 (16.0)	<0.0001
	360	47/48 (97.9)	9/50 (18.0)	<0.0001
	540	47/48 (97.9)	14/50 (28.0)	<0.0001
	720	48/48 (100.0)	15/50 (30.0)	<0.0001
12–23	0	2/50 (4.0)	2/63 (3.2)	1.000
	56	50/50 (100.0)	2/63 (3.2)	<0.0001
	180	50/50 (100.0)	18/63 (28.6)	<0.0001
	360	50/50 (100.0)	19/63 (30.2)	<0.0001
	540	50/50 (100.0)	22/63 (34.9)	<0.0001
	720	48/50 (96.0)	27/63 (42.9)	<0.0001
24–35	0	5/43 (11.6)	12/52 (23.1)	0.184
	56	43/43 (100.0)	12/52 (23.1)	<0.0001
	180	43/43(100.0)	29/52 (55.8)	<0.0001
	360	42/43 (97.4)	31/52 (59.6)	<0.0001
	540	43/43 (100.0)	31/52 (59.6)	<0.0001
	720	43/43 (100.0)	35/52 (67.3)	<0.0001
36–71	0	5/20 (25.0)	13/24 (54.2)	0.047
	56	20/20 (100.0)	13/24 (54.2)	<0.0001
	180	20/20 (100.0)	19/24 (79.2)	0.031
	360	20/20 (100.0)	19/24 (79.2)	0.031
	540	20/20 (100.0)	18/24 (75.0)	0.016
	720	19/20 (95.0)	18/24 (75.0)	0.071
Total	0	12/161 (7.5)	28/189 (14.8)	0.028
	56	161/161 (100.0)	28/189 (14.8)	<0.0001
	180	160/161 (99.4)	74/189 (39.2)	<0.0001
	360	159/161 (98.8)	78/189 (41.3)	<0.0001
	540	160/161 (99.4)	85/189 (45.0)	<0.0001
	720	158/161 (98.1)	95/189 (50.3)	<0.0001

^a^Neutralization antibody titre ≥1:8.

^b^Positive rate – Number (%)

**Fig. 3:**
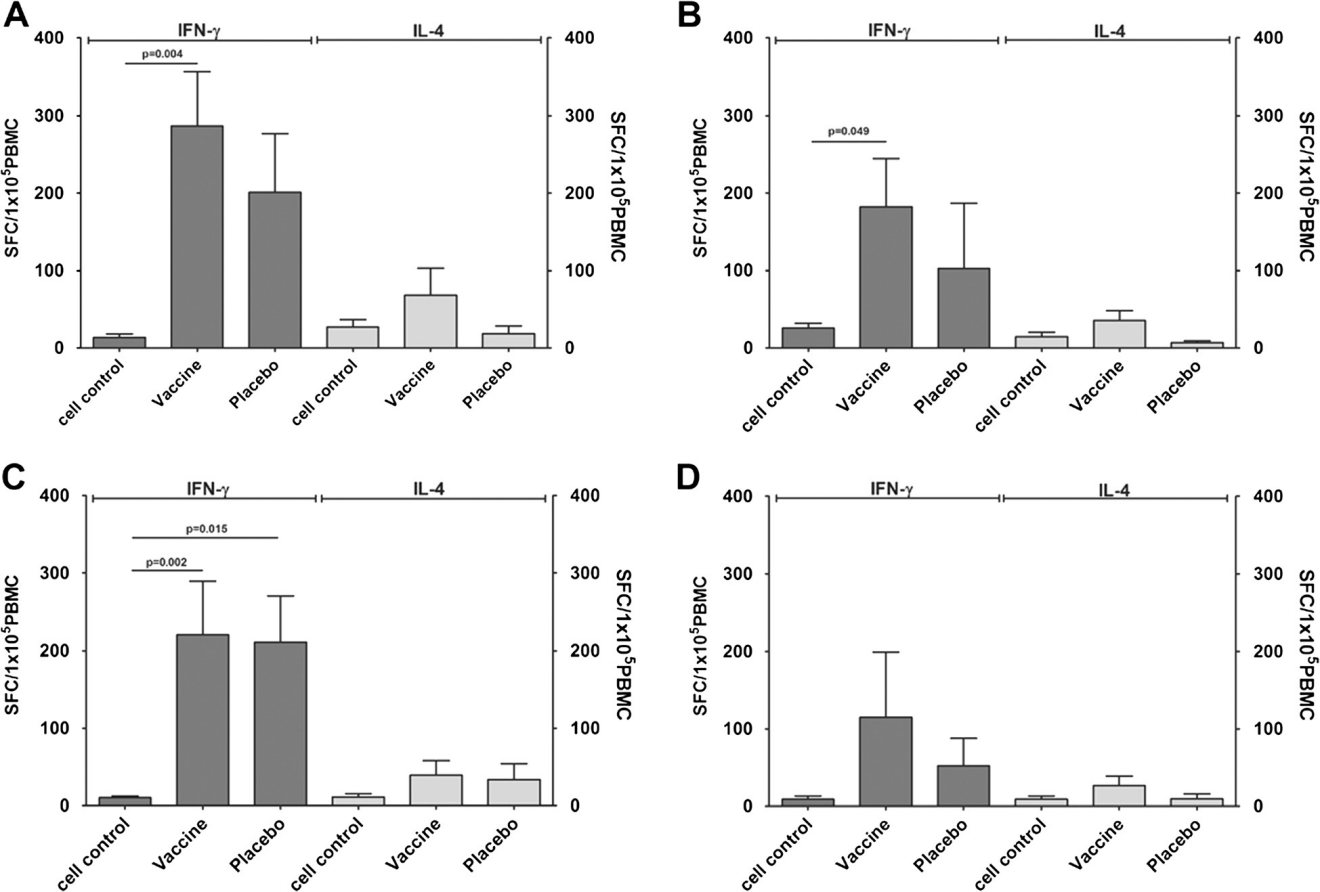
EV71 antigen stimulated specific IFN-γ and IL-4 responses in peripheral blood mononuclear cells from volunteers immunized with either the vaccine or placebo. Blood samples from 40 participants from among the 350 subjects who provided a full series of blood samples within the 2-year follow-up period were obtained at 720 days after immunization to evaluate IFN-γ and IL-4 production. These children were 6–11 months old (**a**) (five individuals in the vaccine group and five in the placebo group), 12–23 months old (**b**) (five individuals in the vaccine group and five in the placebo group), 24–35 months old (**c**) (five individuals in the vaccine group and five in the placebo group), and 36–71 months old (**d**) (five individuals in the vaccine group and five in the placebo group). The mean levels of cytokines are shown above the bar

The capacity to elicit immune memory in children is also an important indicator of the clinical utility of a vaccine [29]. Previous animal experiments and phase II trials primarily measured the effective immune memory, based on the IFN-γ-specific ELISPOT response of PBMCs to stimulation with EV71 antigen in infant populations within 2 months of immunization with two doses of an inactivated EV71 vaccine [30, 31]. To evaluate the variations in the immune memory elicited in the immunized population over an extended period of time, specific anti-IFN-γ and IL-4 ELISPOT assays were performed on PBMCs collected at 720 d.p.i. from 40 (10 from each age group, with five from the immunized group and five from the placebo group) of the 350 subjects to determine the response to stimulation with a specific EV71 antigen using a synthesized VP1 peptide. The results showed significant IFN-γ-specific ELISPOT responses to EV71 antigen stimulation in the immunized children aged 6–11 months, 12–23 months, and 36–71 months (Fig. 3). The IL-4-specific ELISPOT responses in these groups were also significantly greater than those in the placebo control groups (Fig. 3). However, the IFN-γ- and IL-4-specific responses in the children aged 36–71 months were unexpectedly lower than those in the other immunized groups, probably reflecting the limited sample size (only five samples from each group). Therefore, these findings require further investigation. Notably, in addition to the unexpected responses in the immunized group aged 24–35 months, an enhanced IFN-γ response was observed in the placebo control group, induced by an unidentified cause (Fig. 3c). Although further clarification of these unexpected events is required, these data suggest the maintenance of an immune memory response and the induction of an antibody response by the vaccine, which might have been affected by virus circulating in the environment, through an as-yet-unknown pathway [32].

**Fig. 4:**
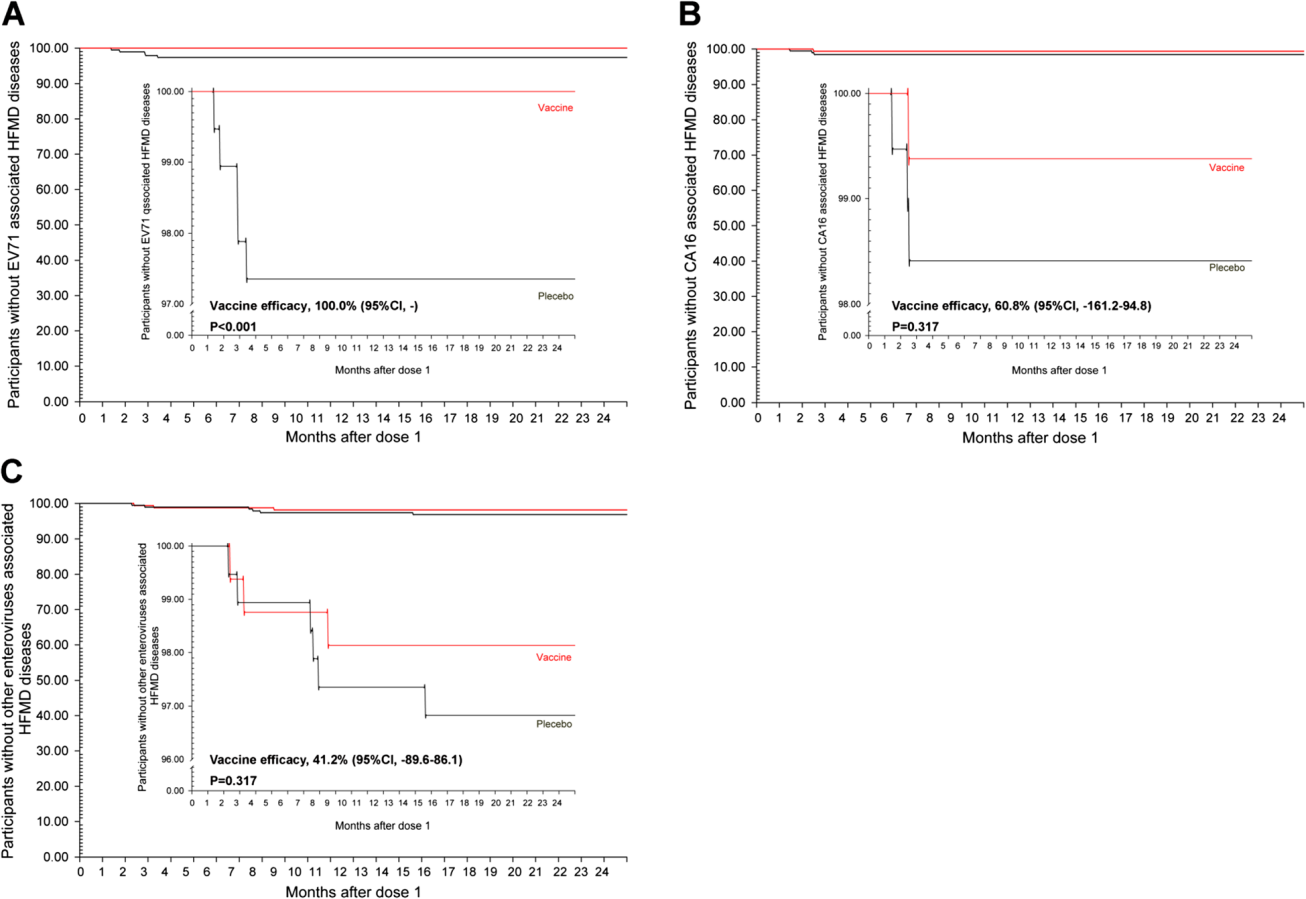
Cumulative cases of hand, foot, and mouth disease (HFMD) reported in 350 participants. A total of 350 participants (161 individuals in the vaccine group and 189 in the placebo group), who provided a full series of blood samples (at all the sampling points) within the 2-year study period, were observed during the surveillance period. The cumulative curves for the cases of HFMD induced by enterovirus 71 (EV71), coxsackievirus A16 (CA16), and other enteroviruses were estimated as a percentage among these participants with Kaplan–Meier survival curves during the period from the receipt of the first dose until 24 months thereafter. The inset shows the same data on an enlarged y-axis. Differences in the distributions of cases of HFMD among the individuals who received the placebo and those who received the vaccine were evaluated with a log-rank test. **a** Cumulative curve for the cases of HFMD induced by EV71. **b** Cumulative curve for the cases of HFMD induced by CA16. **c** Cumulative curve for the cases of HFMD induced by other enteroviruses

These results are consistent with the clinical protective efficacy of this vaccine. The cumulative clinical cases in 350 participants included five EV71-infected subjects (in the placebo group) (Fig. 4a), four CA16-infected subjects (one in the vaccine group and three in the placebo group), and nine subjects infected with other enteroviruses (three in the vaccine group and six in the placebo group; Fig. 4b,c). Including the five described above, totally 15 EV71-infected cases (all in placebo group) were observed in 1,100 subjects, along with 11 CA16-infected cases (three in vaccine and eight in placebo) and 29 other enterovirus-infected cases (12 in vaccine and 17 in placebo; Additional file 1: Figure S1). These cases were identified with clinical diagnoses and etiological RT–PCR analyses.

**Fig. 5:**
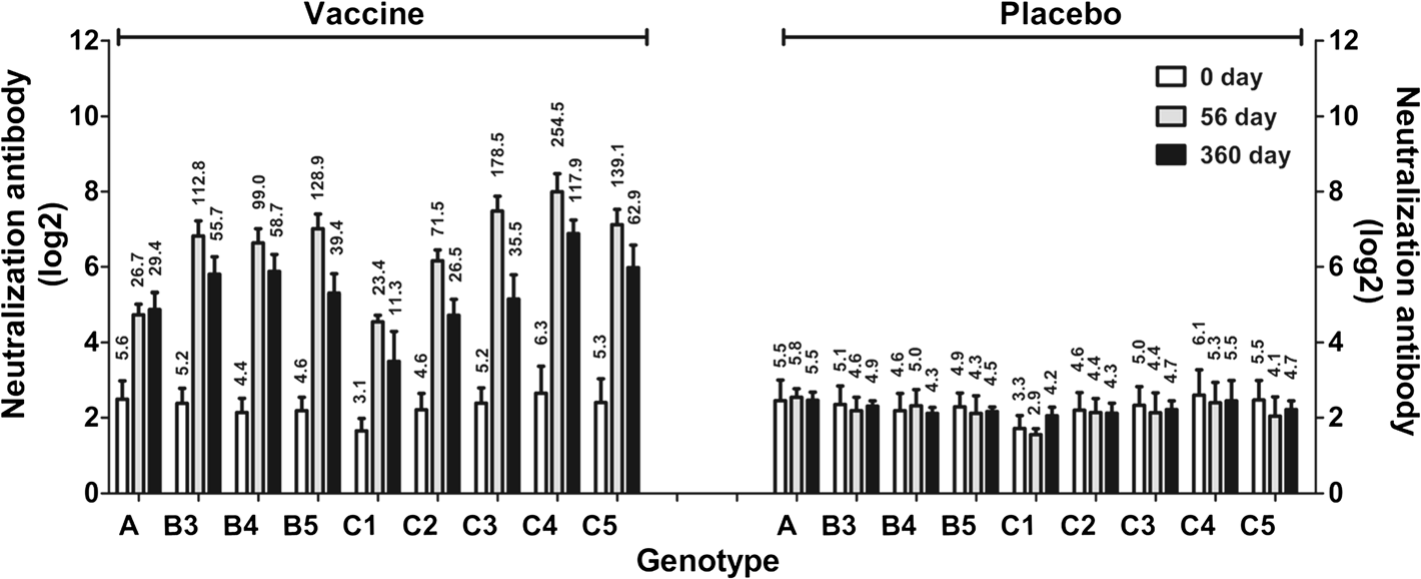
Cross-neutralizing reactivity of different viral strains of various genotypes to sera taken from 160 individuals. Serum samples from 160 individuals (40 individuals in each age group; 20 individuals in the vaccine group and 20 in the placebo group), from among the 350 subjects who provided a full series of blood samples within the 2-year study period, were collected at 0, 56, and 360 days post-immunization for a cross-neutralization assay with different genotypes of enterovirus 71: A, B (B3–B5), and C (C1–C5). The geometric mean titres are shown above the bar. The bar indicates the 95 % confidence interval

These clinical data on the protective effects of the vaccine and our consistent immunological observations, including neutralizing antibody and immune memory responses, collectively demonstrate the effective and sustainable immunogenicity of this vaccine, consistent with the results of our previous clinical trial [8]. Epidemiological studies have shown that the individual circulating EV71 viral strains, including more than 10 genotypes and sub-genotypes, display a great variety of distinctive characteristics in different geographic regions and over different time periods [33, 34]. Recent EV71 candidate vaccines have typically been developed based on the C4 genotype strain circulating throughout mainland China [8–10], but it is unclear whether inactivated candidate vaccines induce immunity with equal efficacy against infections by strains of other genotypes. We randomly selected 160 individuals (40 individuals from each age group: 20 individuals in the vaccine group and 20 in the placebo group) and collected serum samples at 0, 56, and 360 d.p.i. The samples were used in a cross-neutralization assay with nine EV71 strains belonging to genotypes A, B, and C. Of the strains used (A, B3, B4, B5, C1, C2, C3, C4, and C5), all but genotype A were predominant strains circulating across broad regions of Asian-Pacific countries [12–15]. The results showed remarkable cross-neutralizing reactivity to the majority of these strains, with a tendency towards a more dynamic antibody response in the cohorts immunized with this vaccine than in the placebo-treated controls (Fig. 5). Interestingly, all of the sera exhibited similar low neutralizing titres to the genotype C1 strain, although these neutralizing titres were higher than the positive control titre of 1:8 (Fig. 5); the reason for this result is largely unknown. However, the body of evidence presented here and the data of Zhang, Huang, and Mao [35–37] are sufficient to conclude that an inactivated EV71 vaccine derived from one genotype strain could be effectively used to induce adequate protective immunity against infection by most of the predominant circulating EV71 strains.

The present study, which examined a sub-cohort of the population used for a clinical phase III trial of this new viral vaccine, did not include all the subjects in the trial, and importantly, was unblinded at 1 year after vaccination, according to the design requirements. However, this unblinding did not affect the reliability of neutralizing antibody results for these subjects. This study also included a comparison of the protective efficacy of the vaccine in the immunized and placebo-treated groups. The observation of a lower neutralizing capacity against a genotype C1 strain that has been detected sporadically in Asian populations [38] was perplexing, especially as the amino acid sequence of the VP1 protein of this strain is similar to that of the other genotypes. The enhanced IFN-γ ELISPOT response to stimulation with an EV71 antigen in the PBMCs from 2- to 3-year-old children in the placebo control group relative to the response in the vaccinated group remains unexplained. The 2-year observation period in the present study was too short since more information about the immunogenicity of this new vaccine is required.

## Conclusions

This study provides data on the systemic immune response induced by this EV71 vaccine, including the clinical protective efficacy against EV71 infection, the specific immune memory, and the neutralizing antibody response, particularly with respect to initial interactions with various pandemic EV71 strains. Therefore, our results support the use of this vaccine as an immunological strategy for children in the near future.

## Additional file

Additional file 1:
**Detailed information for Method section (including the description of virus strains, summary of long-term phase III clinical trail, the ethics and protocol of long–term observation of phase III clinical trial, the consent form and additional supplemental data.** Figure S1 legendA total of 1,100 participants, receiving either the vaccine or placebo, were observed during the surveillance period. The cumulative curves for the cases of HFMD induced by enterovirus 71 (EV71), coxsackievirus A16 (CA16), and other enteroviruses were estimated as a percentage among these participants with Kaplan–Meier survival curves during the period from the receipt of the first dose until 24 months thereafter. The inset shows the same data on an enlarged y-axis. Differences in the distributions of cases of HFMD among the individuals who received the placebo and those who received the vaccine were evaluated with a log-rank test. (A) Cumulative curve for the cases of HFMD induced by EV71. (B) Cumulative curve for the cases of HFMD induced by CA16. (C) Cumulative curve for the cases of HFMD induced by other enteroviruses. (PDF 2302 kb)

## Abbreviations

CA16: Coxsackievirus A16
ELISPOT: Enzyme-linked immunospot
EV71: Enterovirus 71
GMTs: Geometric mean titres
HFMD: Hand, foot, and mouth disease
IFN-γ: Interferon γ
IL-4: Interleukin 4
PBMCs: Peripheral blood mononuclear cells
