# Supplementary material for: Immunity and clinical efficacy of an inactivated enterovirus 71 vaccine in healthy Chinese children: a report of further observations
Source: BMC Med. 2015 Sep 17;13:226. doi: 10.1186/s12916-015-0448-7 (PMC4574357; doi:10.1186/s12916-015-0448-7)
Supplement: Additional file 1: — Detailed information for Method section (including the description of virus strains, summary of long-term phase III clinical trail, the ethics and protocol of long–term observation of phase III clinical trial, the consent form and additional supplemental data. Figure S1 legendA total of 1,100 participants, receiving either the vaccine or placebo, were observed during the surveillance period. The cumulative curves for the cases of HFMD induced by enterovirus 71 (EV71), coxsackievirus A16 (CA16), and other enteroviruses were estimated as a percentage among these participants with Kaplan–Meier survival curves during the period from the receipt of the first dose until 24 months thereafter. The inset shows the same data on an enlarged y-axis. Differences in the distributions of cases of HFMD among the individuals who received the placebo and those who received the vaccine were evaluated with a log-rank test. (A) Cumulative curve for the cases of HFMD induced by EV71. (B) Cumulative curve for the cases of HFMD induced by CA16. (C) Cumulative curve for the cases of HFMD induced by other enteroviruses. (PDF 2302 kb) [file 12916_2015_448_MOESM1_ESM.pdf]

Supplemental material

TABLE OF CONTENTS

	Pages
**Methods**	
Virus stains	2
Summary of long-term phase III clinical trail	3-4
The ethics of long-term observation of phase III clinical trial	5-7
The protocol of long-term observation of phase III clinical trial (Chinese)	8-22
The protocol of long-term observation of phase III clinical trial (English)	23-42
Consent form (Chinese)	43-44
Consent form (English)	45-47
**Supplemental data**	
Supplemental Table 1	48
Supplemental Figure 1	49-50

Methods

Virus stains

A total of 9 strains of EV71 virus were used in this assay.

Genotype	Name	Genebank No	Isolated area	Isolated time
C1	9522	AY258300	Malaysia	2003
C2	8M/6/99	AY126012	Malaysia	1999
C3	001-KOR-00	AY125966	Korea	2000
C4	FY-23	EU812515	China	2004
C5	VN5559	AM490158	Vietnam	2005
B3	13903	AY207648	Malaysia	1997
B4	A10/4	AF376067	Malaysia	2000
B5	15431	NA*	Malaysia	2006
A	BrCr	U22521	USA	1970

*NA mean not available.

Study designed

A long-term Study of Inactivated EV71 Vaccine (Human Diploid Cell, KMB-17) in Chinese Infants and Children. 1,100 children from 12,000 healthy volunteers qualified all injection 350 people at the age of 6-11 months, 350 people at the age of 12-23 months, 300 people at the age of 24-35 months and 100 people at the age of 36-71 months, with randomly assigned to receive either the vaccine or the placebo at a ratio of 1:1 within each age group, are selected for whole blood collection of 2-3ml at day 0 (the day before the first injection), 56 (28 days after the second injection), 180, 360, 540 and 720 after complete the qualified all injections. Serum is separated and used for serum neutralization antibody detection. The seroconversion rate and GMT for 56, 180, 360, 540 and 720 day-post immunization is obtained.

Clinicaltrail number: NCT01569581

(http://clinicaltrials.gov/ct2/show/NCT01569581?term=NCT01569581&rank=1)

The other details of the phase III clinical study and statistical analysis were uploaded in NEJM (Supplementary Appendix, 2014, 370: 829-37).

Briefly:

The study was designed as multiple centers, randomize, double-blind, 1:1 ratio placebo-controlled clinical study. The vaccine used for clinical trial is EV71 inactivated vaccine 100U (aluminum adjuvant), placebo used in the study is vaccine PBS diluent containing aluminum hydroxide. 12,000 healthy subjects are recruited, in which there are 3,500 of 6-11 months, 3,500 of 12-23 months, 3,000 of 24-35 months, and 2,000 of 36-71 months. Under each age group, subjects are randomly divided into

two groups that inject 100U (aluminum adjuvant) dosage and placebo respectively. Injection program: 0, 28-day injection procedure is used for 100U (aluminum adjuvant) dosage clinical study vaccine or placebo injection.

Ethics

声明：本伦理委员会组成和工作程序符合 GCP 原则和国家相关法律法规

批准证明书

GXIRB
广西伦理审查委员会
IRB00001594　FWA00001359
广西疾病预防控制中心
IORG0001186
中国广西南宁市金洲路 18 号，　530028

批准日期：2013 年 3 月 20 日

项目名称：肠道病毒 71 型灭活疫苗（人二倍体细胞）抗体免疫持久性研究

广西现场负责人：　莫兆军　　电话：0771—2518780

申办者：中国医学科学院医学生物学研究所

批准项目文件：

临床研究方案、知情同意书

审阅内容包括：

1. 临床研究方案（抗体免疫持久性）　版本号：1.0 版本日期：2013 年 3 月 15 日
2. 知情同意书（抗体免疫持久性）　版本号：1.0 版本日期：2013 年 3 月 18 日

2013 年 3 月 20 日广西伦理审查委员会召开会议，在本次会议上对上述所列项目内容进行了有关伦理方面的审查。通过审查，认为上述文件符合中国的伦理，可以用于中国医学科学院医学生物学研究所的"肠道病毒 71 型灭活疫苗（人二倍细胞）抗体免疫持久研究"项目，会议对上述文件的审查进行了投票决定，参加会议的委员一致通过，并批准上述方案在广西进行研究。

谢永洪，主席　　　　2013 年 3 月 20 日
（日　期）

广西伦理审查委员会成员名单
Guangxi Institutional Review Board Composition

伦理审查委员会名称：广西伦理审查委员会<br>NAME OF IRB: GUANGXI IRB	伦理审查委员会编号：<br>IRB: 00001594 FWA: 00001359

Review Date： 2013 年 3 月 20 日

成员职称/姓名<br>Member Title and Name	单位<br>Work Unit	职业<br>Occupation (position)	学历(如有)<br>Qualification (If applicable)	男/女<br>Male/ Female	在参加方案讨论的委员处打(√)<br>Tick (√)if member present when protocol reviewed
主席 谢永洪<br>Chairman<br>Yonghong XIE	广西疾病预防控制中心<br>Guangxi CDC	教授<br>Professor	研究生<br>Postgraduate	男<br>M	√
秘书 刘帅凤<br>Secretary<br>Shuaifeng LIU	广西疾病预防控制中心<br>Guangxi CDC	药师<br>Pharmacist	研究生<br>Postgraduate	女<br>F	√
杨进业<br>Jinye YANG	广西疾病预防控制中心<br>Guangxi CDC	主任医师<br>Doctor	研究生<br>Postgraduate	男<br>M	√
戴庆瑄<br>Qingxuan DAI	广西社会科学院<br>Guangxi Academy of Social Science	社会工作者<br>Social worker	研究生<br>Postgraduate	女<br>F	
韩宇玲<br>Yuling HAN	伟宁律师事务所<br>Wei Ning Law Office	律师<br>Lawyer	研究生<br>Postgraduate	女<br>F	√
李荣成<br>Rongcheng LI	广西疾病预防控制中心<br>Guangxi CDC	教授<br>Professor	研究生<br>Postgraduate	男<br>M	
陈 杰<br>Jie CHEN	广西疾病预防控制中心<br>Guangxi CDC	主任医师<br>Doctor	研究生<br>Postgraduate	男<br>M	
刘 伟<br>Wei LIU	广西疾病预防控制中心<br>Guangxi CDC	主任医师<br>Doctor	研究生<br>Postgraduate	女<br>F	√
李慕军<br>Mujun Li	广西医科大学一附院<br>Hospital of Guangxi Medical University	主任医师<br>Doctor	研究生<br>Postgraduate	女<br>F	
林新勤<br>Xinqin LIN	南宁市疾病预防控制中心<br>Nanning CDC	主任医师<br>Doctor	研究生<br>Postgraduate	男<br>M	√
李 进<br>Jin LI	广西卫生职业技术学院<br>Guangxi Health College of Technology	教授<br>Professor	研究生<br>Postgraduate	女<br>F	
吕 炜<br>Wei LU	广西疾病预防控制中心<br>Guangxi CDC	副主任医师<br>Doctor	研究生<br>Postgraduate	男<br>M	√
谢庆玲<br>Xie Qinglin	广西区人民医院<br>Guangxi Provincial People's Hospital	儿科主任医师<br>Pediatrician	研究生<br>Postgraduate	女<br>F	√
龚 健<br>Gong Jian	广西疾病预防控制中心<br>Guangxi CDC	主任医师<br>Doctor	研究生<br>Postgraduate	女<br>F	√
于 丽<br>Li Yu	广西疾病预防控制中心<br>Guangxi CDC	助理研究员<br>researcher	研究生<br>Postgraduate	女<br>F	
蒙晓宇<br>Meng XiaoYu	广西疾病预防控制中心<br>Guangxi CDC	医师<br>Physician	研究生<br>Postgraduate	男<br>M	√

Ethical approval

GXIRB
Guangxi Ethics Committee
IRB00001594 FWA00001359
Guangxi Centers for Disease Control and Prevention
IORG0001186
No.18 Jinzhou Road, Nanning, Guangxi, China, 530028

Approved Date: March 20th, 2013
Project Title: Long-term effect on immunity and the efficacy of the inactivated enterovirus 71 vaccine (human diploid cell)
Principal Investigator: Zhaojun Mo Tel: 86-0771-2518724
Sponsor: Institute of Medical Biology, Chinese Academy of Medicine Sciences

Approved project includes:
The protocol of clinical study and the informed consent

A review includes:

1. The protocol of long-term study	Rev 1.0, Date:20130315
2. The informed consent	Rev 1.0, Date:20130318

The meeting has been convened by Guangxi Ethics Committee, on March 20th, 2013. The items listed above have been reviewed and examined at this meeting. The experts thought that these documentations accorded with Chines ethical requirement and were carried out into the long-term study of the inactivated enterovirus 71 vaccine (human diploid cell), which was applied by Institute of Medical Biology, Chinese Academy of Medicine Science. The items have been approved by all ethical experts at this meeting. The project would be carried out in Guangxi Proviouse.

(Chirman)____________ (Date)____________

项目名称：肠道病毒 71 型灭活疫苗（人二倍体细胞）
抗体免疫持久性研究

研究产品名称：肠道病毒 71 型灭活疫苗（人二倍体细胞）

申办方：中国医学科学院医学生物学研究所

批 件 号：2010L05009

制品分类：预防用生物制品 I 类

方案编号：201216302-C

方案版本日期：2013 年 3 月 15 日　版本号：V1.0

临床研究负责单位：广西壮族自治区疾病预防控制中心

临床研究地点：　广西壮族自治区

临床研究时间：　2013 年 3 月至 2014 年 6 月

肠道病毒 71 型灭活疫苗（人二倍体细胞）抗体免疫持久性研究

主要研究者：

姓名： 莫兆军
性别： 男
职称： 副主任医师
职务： 广西疾病预防控制中心疫苗临床研究所副所长
单位： 广西壮族自治区疾病预防控制中心
地址： 广西壮族自治区南宁市金洲路 18 号
联系电话：0771-2518780
传真：0771-2518986
E-mail：mozhj@126.com

临床研究监查员：

姓名：黄林雄
单位：北京思睦瑞科科技有限公司
地址：广西南宁市桃源路 43 号 710 室
电话：18677085876
传真：0771-5301283
E-mail：lindon.huang@simoonrecord.com

研究者声明

我认真学习了此方案，包括附加信息，我会在遵循伦理要求的前提下，严格按照方案要求实施本次临床试验研究。

主要研究者签名：莫兆军　　日期：2013 年 3 月 15 日

版本号: 1.0　　版本日期：20130315

同意

2013年3月20日

目 录

1. 研究背景 5

2. 研究现场及研究合作单位 6

2.1. 研究现场 6

2.2. 研究合作单位 6

3. 研究目的和终点 6

3.1. 研究目的 6

3.2. 研究终点 7

4. 研究设计 7

4.1. 预计受试者在研究中的大致时间 7

4.2. 研究期限 7

4.3. 预期受试者数 7

5. 观察对象的选择 7

5.1. 排除标准 8

5.2. 退出与中止标准 8

6. 研究治疗 8

6.1. 研究疫苗 8

6.2. 研究中允许的治疗 8

7. 研究步骤 8

7.1. 入组访视 9

7.2. 第6个月及第12个月访视 9

8. 血样采集 9

8.1. 采血要求 9

8.2. 血样编码 10

9. 数据分析/统计学方法 10

10. 临床研究的质量保障和监控 10

10.1. 研究者培训 10

10.2. 监查员 10

10.2.1 要求 10

10.2.2 职责 11

10.3. 观察对象安全性 11

10.4. 标本管理 12

同意
2013年 3月 2 0[illegible]

10.5.　仪器设备校验、体温计及玻璃器皿标化 12
10.6.　临床研究数据管理 12
10.7.　研究资料提供和保存 12
10.8.　协议和临床研究报告 13

11.　时间表 13

12.　伦理委员会 13
12.1.　审核、批准应用于临床研究的文件 13
12.2.　实施监督 13
12.2.1 知情同意 13
12.2.2 保密性 14
12.2.3 潜在危险和危险最小化 14

13.　不良事件处理 14

14.　附录 15

15.　参考文献 15

同意 2013年 3月 20日

1. 研究背景

手足口病（HFMD）为普遍流行于婴幼儿及学龄前儿童群体中的常见病毒性传染病，其通常在大多数患者个体所表现临床症状并不十分严重，主要为轻微的感冒症状，并伴有手掌、足心及口腔粘膜的疱疹，其病程通常局限于 5-7 天内。在 HFMD 发病群体中，有部分的患者可以出现严重的临床症状。其包括脑炎、脑干脑炎、无菌性脑膜炎、脊髓炎，以及由此神经系统综合症所引起的神经源性肺水肿及肺出血，或是心肺功能衰竭。这类表现严重的神经系统综合症及心-肺功能衰竭综合症可引起较高的死亡率。HFMD 的病原学分析早已证明，引起该疾病发生的病原主要为人类肠道病毒，其包括 EV71、CoxA16、CoxB3 等等，但从病毒分离及病人血清抗体检查看，EV71 是引起 HFMD 的主要病原。近二十年来，HFMD 成为人们关注的病毒性传染病。2008 年安徽省阜阳市出现的数十例重症死亡的病例，有 60%以上是由 EV71 引起，绝大多数的 HFMD 重症病人都与 EV71 感染相关。

中国医学科学院医学生物学研究所生产的肠道病毒 71 型灭活疫苗系用人二倍体细胞转瓶培养工艺培养 C4 基因型的 FY-23K-B 株 EV71 病毒，经灭活、纯化制成。用于预防 EV71 感染引起的婴幼儿手足口病。经国家食品药品监督管理局依据《中华人民共和国药品管理法》和《药品注册管理办法》（试行）审查，该制品符合药品注册的有关规定，以 2010L05009 号《药物临床试验批件》批准该制品进行临床试验。该制品于 2011 年 1 月至 6 月完成Ⅰ临床试验确定安全性后，开展Ⅱ期临床试验对不同剂量和不同程序进行探索，确定了Ⅲ期临床试验的免疫剂量。

2012 年 2 月，受中国医学科学院医学生物学研究所委托，广西壮族自治区疾病预防控制中心作为负责单位，根据国家食品药品监督管理局《药品注册管理办法》、《药物临床试验质量管理规范》（GCP）及《疫苗临床试验技术指导原则》的要求在在广西桂林市兴安等七县开展了一项多中心、随机、双盲、安慰剂对照的Ⅲ期临床研究，以评价疫苗的免疫效力、安全性和免疫原性。研究对象为 12000 名 6-71 月龄健康婴幼儿及儿童，受种者以 1:1 的比率被随机分组，按照 0,28 天的免疫程序接种疫苗或者安慰剂。其中 1100 个对象为免疫原性亚组，分别在疫

同意
永[illegible]
洪[illegible]
2013年 3月 20[illegible]

苗接种前、全程接种后 28 天及 180 天采集静脉血进行 EV71 抗体检测。临床研究结果表明，试验疫苗预防由肠道病毒 71 型引起的手足口病的疫苗效力为 97.3%（95% CI：92.6%,99.0%），预防由肠道病毒 71 型引起的重度手足口病的疫苗效力为 100%，达到方案设计的效力目标。试验疫苗组易感受种者全程免疫后 28 天抗体阳转率达到 100%，GMT 为 224.37，安慰剂组分别为 4.31%，抗体 GMT 为 7.44，试验疫苗组抗体阳转率和 GMT 显著高于安慰剂组。全程免疫后 180 天，试验疫苗组抗体阳性率和抗体 GMT 仍维持在 95.81%和 1：118.0。该疫苗具有良好的安全性和免疫原性。

为了进一步观察该疫苗接种后的长期免疫原性，设计了本次随访研究，拟对原 EV71 疫苗Ⅲ期临床研究中的免疫原性亚组对象的疫苗免疫后 1-2 年的抗体水平进行评价。

2. 研究现场及研究合作单位

2.1. 研究现场

选定原 EV71 灭活疫苗Ⅲ期临床研究中入组了免疫原性亚组受种者的永福县、灵川县、荔浦县、兴安县作为本次临床研究的现场。

2.2. 研究合作单位

由中国食品药品检定研究院负责本临床研究 EV71 病毒血清中和抗体检定。

3. 研究目的和终点

3.1. 研究目的

主要目的：

评价接种两剂 EV71 疫苗后第 12 个月、18 个月和 24 个月，疫苗诱导的血清 EV71 中和抗体的抗体滴度。

次要目的：

评价接种两剂 EV71 疫苗后第 12 个月、18 个月和 24 个月，疫苗诱导的血清 EV71 中和抗体的血清阳性率。

同意 永谢 共 2013年 3月 2[illegible]

3.2. 研究终点

研究终点为血液样本中的血清 EV71 中和抗体的抗体滴度（GMT）和阳性率。

4. 研究设计

招募参与了中国医学科学院医学生物学研究所 EV71 灭活疫苗（人二倍体细胞）III期临床研究并完成观察的免疫原性亚组受种者作为观察对象。需在入组时、入组后 6 个月及 12 个月分别完成一次血样采集，每次 2-3ml。不研究不包含疫苗接种。

4.1. 预计受试者在研究中的大致时间

观察对象在研究中的参与时间大约为 3 天。

4.2. 研究期限

研究将从 2013 年 3 月下旬开始。研究结束时间是获得所有血清学数据的日期（数据库锁定），预计为 2014 年 6 月。

4.3. 预期受试者数

原 EV71III期临床研究中完成观察的免疫原性亚组对象均将被邀请参加本研究。

5. 观察对象的选择

以监护人知情同意、自愿参加为原则。

入选标准

■原 EV71III期临床研究中完成全程安全性及免疫原性观察的免疫原性亚组对象；

■监护人了解本次研究的内容和要求，自愿参加此项研究并签署知情同意书；

■按方案要求能接受三次采集血液标本进行 EV71 中和抗体检查。

同意
永洪
2013年 3月 20日

5.1. 排除标准

符合下列任何一项的观察对象将不能入选本研究。

■在完成原 EV71Ⅲ期临床研究后，接种了已注册的或研究性质的 EV71 疫苗；

■在原 EV71Ⅲ期临床研究中曾患经实验室确认 EV71 感染的手足口病；

■在入选本研究时，正在参加其他研究；

■根据研究者判断，观察对象有其他严重的急性或慢性疾病或精神情况或实验室检查异常，导致受试者参加研究的相关风险显著增加，或可能干扰研究结果的解释，而不适合参加本研究。

5.2. 退出与中止标准

观察对象出现以下任意一种情况时，将提前中止临床观察：

■ 监护人要求退出临床观察；

■ 观察对象的健康状况不允许其参加本临床观察；

■ 观察对象接种了已注册的 EV71 疫苗；

■ 观察对象在观察期间发生经实验室确认 EV71 感染的手足口病；

■ 观察对象出现任何异常临床表现，由研究者决定其是否提前中止临床观察。

6. 研究治疗

6.1. 研究疫苗

不研究不包含疫苗接种。

6.2. 研究中允许的治疗

允许用疫苗：本临床研究期间不允许进行其它疫苗临床研究或接种其他研究性质的疫苗。可以进行常规疫苗及应急疫苗如狂犬病或破伤风等疫苗的接种。

允许用药：在研究期间，观察对象如出现不良事件，应允许必要的药物治疗。

7. 研究步骤

研究步骤详见研究流程表。

同意
2013年 3月 20日

表 1　研究流程

工作内容	访视时间		
	入组访视	第 6 个月	第 12 个月
知情同意	×		
审核入组/排除标准	×		
采集血样	×	×	×

注：采血窗口期设定为一个月。

7.1.　入组访视

7.1.1 对观察对象监护人详细讲解《EV71 灭活疫苗免后抗体持久性研究知情同意书》，获得志愿者监护人的知情同意，监护人、研究医生共同签署《EV71 灭活疫苗免后抗体持久性研究知情同意书》，一式两份，双方各执一份。

7.1.2 研究者对志愿者进行入组/排除标准审核，判定是否入组及是否可以承受采血。

7.1.3 给入组的志愿者采集 2-3ml 静脉血并记录《样本采集记录表》。

7.2.　第 6 个月及第 12 个月访视

研究者对观察对象进行可以承受采血的判断后对其进行采血，每次 2-3ml，并记录《样本采集记录表》。

8.　血样采集

8.1.　采血要求

将采集的观察对象的 2-3ml 静脉血置于非抗凝管并进行血清分离以避免发生溶血现象。将分离出的血清分装于送检和留样两个血清管中，其中用于送检的血清至少保证 0.5ml，剩余的血清则作为备份保存于留样管中。血清管上需粘贴血清编码标签标识，并保存在-20℃下。如果不能迅速将血清样本冷冻，则应先将其冷藏，但最好在 24 小时内冷冻。送检血清将分批次送至中国食品药品检定研究院进行 EV71 病毒中和抗体检测。

采集血液及分离血清全过程应尽量保证无菌操作，需送检的血清冻存于-20℃以下，运输血清标本时应保证温度低于 4℃。

同意
永洪

2013年 3月 2 0日

8.2. 血样编码

第一次血样：原研究编号-1

第二次血样编号：原研究编号-2

第三次血样编号：原研究编号-3

示例：原研究编号为 060001 的观察对象的三次采血编号分别为 060001-1、060001-2、060001-3。

9. 数据分析/统计学方法

具体的分析内容参见统计分析计划（SAP）和/或临床研究报告。内容包括缺失的数据，如果适用，还包括未使用的和伪造的数据的详细信息。对统计分析计划的偏离将记录在临床研究报告中。

10. 临床研究的质量保障和监控

10.1. 研究者培训

所有参加本次临床研究人员均为研究者，要求具有医（护）专业资格证书（包括执业医生资格证书、医技人员上岗证等），经以下内容培训考核合格后，参与本项研究。

- 临床观察方案；
- 知情同意书；
- 血样采集记录表等相关表格；
- 静脉血采集及血清分离技术等；
- 《药物临床试验质量管理规范》（GCP）

10.2. 监查员

10.2.1 要求

根据《药品注册管理办法》第三十五条 药物临床研究过程中，申请人应当指定具有一定专业知识的人员监督执行《药物临床试验质量管理规范》。现场监察员负责对临床研究全过程进行监督，确保临床研究内容符合GCP和临床研究方案要求，并在预期内完成。参与本项研究的监察员应熟悉以下内容：

同意 永谢 洪 2013年 3月 20日

- 《药物临床试验质量管理规范》（GCP）及各种法规；
- 临床研究方案；
- 知情同意书；
- 血样采集记录表等相关表格；
- 静脉血采集及血清分离技术等；
- 现场数据的收集。

10.2.2 职责

■临床研究前确认研究单位具有适当的条件，包括人员配备与培训情况，实验室设备齐全、运转良好，具备各种与临床研究有关的检查条件，参与研究人员熟悉临床研究方案中的要求。

■临床研究过程中监查研究者对临床研究方案的执行情况，确认在临床研究前取得所有观察对象的知情同意书，确认入选的受种者合格。

■确认所有数据的记录与报告正确完整，所有表格填写正确。所有错误或遗漏均已改正或注明，经研究者签名并注明日期。

■监督核实临床研究血样按照方案进行采集、分离、储藏、运输，并做相应的记录。

■协助研究者进行必要的通知及申请事宜，向申办者报告临床研究数据和结果；

■定期到达研究现场进行监察并及时（3天内）向申办单位和临床研究负责单位主要研究者提出书面监督工作报告，报告应述明监查日期、时间、监查员姓名、监查的发现等。清楚如实记录研究者未能做到的随访、未进行的临床研究、未做的检查，以及是否对错误、遗漏作出纠正。

■临床研究中出现偏差，由监察员与研究者协商解决，重大事件上报伦理委员会、药品监督管理部门。

10.3. 观察对象安全性

本研究不涉及疫苗接种。血样采集需在乡镇卫生院或疾控中心门诊由有相关资质的护士进行，采血用的注射器或采血针及采血管均为一次性无菌材料，避免交叉感染。研究者在采血前将评估观察对象是否适宜进行血样采集。采血过程中可能观察对象会因疼痛而哭闹，还可能出现采血部位的淤肿等，但研究者将尽量

同意
永洪
2013年 3月 20日

避免。同时在采血过程中研究者会密切关注观察对象情况，当出现不适症状时研究医生会进行及时、良好的治疗。如因本研究引起的严重不良反应，研究方会联系申办方负责支付相应的医疗费用，并根据不良反应的严重程度提供一定的补偿。

10.4. 标本管理

观察对象的血样当天在现场实验室分离血清，尽量避免严重溶血。置-20℃下低温保存，每天监测温度，现场工作全部完成后冷藏运输到指定的检测实验室。

10.5. 仪器设备校验、体温计及玻璃器皿标化

- 恒温培养箱已校验，有效期内，有温度记录
- 冰箱已校验，有效期内，有温度记录
- 体温计等量具已标化
- 采血用注射器为一次性无菌注射器，生产厂家有国家生产许可证，记录批号与效期。

10.6. 临床研究数据管理

按照《药物临床试验质量管理规范》第八章第四十八、第四十九条进行临床研究记录及报告的原始资料应完整保存。临床研究中的任何操作均应及时、准确、完整、规范、真实地记录于相关表格中，不得随意更改，确因填写错误，作任何更正时应保持原记录清晰可辩，由更正者签署姓名和时间。

为保证临床研究数据统计的真实可靠，研究者对所有现场记录进行复核，监查员要对数据记录情况进行定期及不定期的监查，直到表格填写完整，在收回表格前，监查员应仔细核实表格填写内容，复核和监查。审核结果应记录。血样的交接应有文件记录。

所有资料由临床研究负责单位或申办单位委托有关单位或部门专业统计人员进行统计处理。

严重不良事件和达到 3 级的事件应填写个案表、严重不良事件记录表。

10.7. 研究资料提供和保存

- 血清检测结果（检验单位、申办方和研究方）

同意 永洪 谢
2013年 3月 20日

- 临床研究报告（申办方和研究方）
- 临床研究单位委托函、接受函（研究方和申办方）
- 伦理委员会批件（研究方原件，申办方保留复印件）
- 不良事件调查、报告表（SFDA、伦理委员会、研究方和申办方）
- 其他现场原始资料（已签字知情同意书、血样采集登记表等）：现场疾病预防控制中心（保存至临床研究完成后 5 年）

其他资料：

研究负责单位保存血样送检单、血样交接单；实验室检验单位保存血清检测记录及结果。

10.8. 协议和临床研究报告

- 协议书原件：一式 3 份，申办方 2 份、研究方 1 份。
- 临床总结报告（红章）：一式 5 份，研究负责单位省级疾病预防控制中心 1 份存档、申办方 4 份。

11. 时间表

总研究时间：15 个月。

12. 伦理委员会

12.1. 审核、批准应用于临床研究的文件

- 临床研究方案
- 知情同意书样张
- 主要研究者简历

12.2. 实施监督

12.2.1 知情同意

受种者入选的方法和向受种者/监护人/法定代理人提供有关信息资料是否完整、易懂；获取知情同意的方法是否适当。在整个临床研究过程中，伦理委员会要监督是否存在损害受种者伦理方面的问题以及受种者因临床研究受到损害

同意 永洪谢 2013年 3月 20日

时是否得到治疗或补偿和相应的保险措施，同时评价受种者所承受风险的程度。

12.2.2 保密性

确保在临床研究进行和生物样本采集以及报告、出版等条件下受种者个人机密。生物样本只记录 ID 编号或实验室号码。严格限定为主要研究人员才能获得电子或书面复印。

12.2.3 潜在危险和危险最小化

如不良反应认定与血样采集有关，是否按照有关规定及时进行治疗。如果发生威胁生命的事件，有无立即护送到医院治疗和报告的措施。

是否有措施保证在严格的监管下，经过培训、有经验的医务人员按照规定程序进行静脉血收集。以使被采集血样的人经受的痛苦（包括疼痛以及机率很少的静脉穿刺部位局部性感染）减少到最小。

13. 不良事件处理

13.1. 定义

不良事件：是指临床研究中受种者产生的非预期医疗事件，其与疫苗/接种疫苗不一定有因果关系。

不良反应：在按规定剂量和程序接种疫苗过程中，产生非预期或有损害的反应，通常与疫苗接种有关。

严重不良反应：是指与死亡、入院治疗、住院期延长、持续性残疾或无自理能力等与疫苗临床研究有关的有生命危险的事件。包括癫痫、昏迷、手足抽搐、糖尿病酮酸中毒、弥散性血管内凝血、弥散性瘀斑、麻痹或瘫痪、急性精神病、严重抑郁症等。

13.2. 报告和处理

研究者必须使受种者了解接种疫苗后出现任何疾病和异常事件应尽快向研究者报告以及报告的方法。

按照国家有关规定，受种者应到指定的医院接受适当地治疗。研究者应对不良反应进行调查和医学随访，如病史、体检和必要的实验室检查和处理、跟踪，直到事件得以解决，并完成详细的调查表填写和随访记录。调查表内容包括症状、

同意 2013年 3月 20日

体征、诊断和实验室结果。

研究者应在发生严重不良事件24小时内，报告药品监督管理部门、申办方和伦理委员会，申办方按照规定向药品监督管理部门和涉及同一药物的临床研究的其他研究者通报。

14. 附录

1）伦理委员会批准件
2）知情同意书样本
3）临床研究单位情况及资格，主要研究人员简历及授权表
4）临床研究研究方案、方案的修改内容及伦理委员会对修改内容的批准件
5）严重不良事件及主要研究者认为需要报告的重要不良事件的病例报告
6）统计分析报告
7）临床研究报告（血清检测报告）

15. 参考文献

1）《疫苗临床试验技术指导原则》（国食药监注[2004]575 号），国家食品药品监督管理局，2004 年 12 月 3 日。

2）《药物临床试验质量管理规范》（GCP），国家食品药品监督管理局，2003 年 6 月。

3）《结合疫苗质量控制和临床研究技术指导原则》，国家食品药品监督管理局，国食药监注[2005]493 号，第 5 部分。2005 年 10 月 14 日。

Project Name: Antibody Immunity Duration Research of Enterovirus 71 Inactivated Vaccine (Human Diploid Cells)

Product Name: Enterovirus 71 Vaccine (Human Diploid Cells)

The Sponsor: Institute of Medical Biology, Chinese Academy of Medical Sciences

Approval No.: 2010L05009

Product Classification: Prophylactic Biological Product, Class I

Proposal No.: 201216302-C

Proposal Version: March 15, 2013, Version: V1.0

Research Institute: Center for Disease Control and Prevention of Guangxi Zhuang Autonomous Region

Research Location: Guangxi Zhuang Autonomous Region

Research Period: March 2013 to June 2014

Antibody Immunity Duration Research of Enterovirus 71 Inactivated Vaccine (Human Diploid Cells)

Principle investigator:

Name: **Mo Zhaojun**

Gender: Male

Professional Title: Associate chief physician

Post: Vice director of Vaccine Clinical Research Institute of Center for Disease Control and Prevention of Guangxi Zhuang Autonomous Region

Address: 18 Jinzhou Road, Nanning City, Guangxi Zhuang Autonomous Region, PRC

Tel: 0771-2518780

Fax: 0771-2518986

E-mail: mozhj@126.com

Clinical research associate

Name: Huang Linxiong

Unit: Beijing Simoonrecord Science and Technology Ltd.

Address: Room 710, 43 Taoyuan Road, Nanning City, Guangxi Province

Tel: 18677085876

Fax: 0771-5301283

E-mail: lindon.huang@simoonrecord.com

Statement of Investigators

I have carefully learned about this Project, including the additional information. I would like to carry out this clinical experimental research strictly following the Project requirement on the premise of ethical requirement.

Signature of the principle investigator: ____________________Date: ______________

CONTENTS

1. Background......27
2. Research Site and Partners......28
2.1 Research Site......29
2.2 Partners......29
3. Research Purpose and Endpoint......29
3.1 Research Purpose......29
3.2 Research Endpoint......29
4. Research Design......30
4.1 Approximate Time of Researchers under Research......30
4.2 Research Period......30
4.3 Expected Number of Participants......30
5. Selection of Participants......30
5.1 Requirements for Candidate Exclusion......31
5.2 Requirements for Withdrawing and Suspension......31
6. Treatment Research......32
6.1 Vaccine Research......32
6.2 Allowable Treatment in Research......32
7. Research Steps......32
7.1 Visit upon Recruitment......32
7.2 Follow-up Visit in 6th and 12th Months......33
8. Blood Sampling......33
8.1 Requirements for Blood Sampling......33
8.2 Blood Specimen Coding......34
9. Data Analysis/Statistical Methods......34
10. Quality Assurance and Monitoring for Clinical Research......34
10.1 Training for Researchers......34
10.2 Supervisor......35
10.2.1 Requirements......35
10.2.2 Responsibilities......35
10.3 Safety of Participants......36
10.4 Specimen Management......37

10.5 Instrument Calibration and Thermometer & Glassware Standardization.............37
10.6 Clinical Research Data Management...37
10.7 Supply and Preservation of Research Data 38
10.8 Agreement and Clinical Research Report...39
11. Timetable...39
12. Ethics Committee 39
12.1 Auditing and Approving Documents for Clinical Research 39
12.2 Supervision over Implementation..39
12.2.1 Informed Consent..39
12.2.2 Confidentiality...40
12.2.3 Potential Dangers and Minimization Thereof 40
13. Adverse Event Treatment 40
13.1 Definition...40
13.2 Notification and Treatment 41
14. Appendixes..41
15. References 42

1. Background

Hand-foot-and-mouth Disease (HFMD) is a common viral infectious disease that is generally epidemic among infants and preschoolers. For most patients, individual clinical symptoms are relatively light, mainly reflected by flu symptoms accompanied with herpes on palm, sole centre and oral mucosa. Its duration is usually 5 to 7 days. Among HFMD patients, some shows severe clinical symptoms: encephalitis, brain stem encephalitis, aseptic meningitis, myelitis, neurogenic pulmonary edema and pneumorrhagia caused by neurological syndromes, or cardiorespiratory failure. Such severe neurological syndrome and cardiorespiratory failure may cause higher mortality rate. Etiological analysis has proven that HFMD is mainly caused by human enteroviruses, including EV71, CoxA16 and CoxB3; however, learnt from virus isolation and serum antibody inspection test, EV71 is the major pathogen. In the past two decades, HFMD has become the focus of viral diseases. For instance, among dozens of server cases and deaths occurred in Fuyang City, Anhui Province in 2008, more than 60% were caused by EV71 and the vast majority of HFMD critical patients suffered from EV71 infection.

Enterovirus 71 inactivated vaccine was developed by Institute of Medical Biology, Chinese Academy of Medical Sciences by culturing FY-23K-B strains (subtype C4) in spinner flask, and then inactivating and purifying them, in order to the prevention and control of EV71 epidemics. Upon the examination of China Food and Drug Administration in accordance with *Drug Administration Law of the People's Republic of China* and *Administration Measures for Drug Registration (Trial)*, the product complies with relevant regulations of drug registration and is approved for clinical trial with *Drug Clinical Trial Approval* (Approval No.: 2010L05009). In Phase I, the safety of the product was determined by clinical trial from January to June 2011, and in Phase II, clinical trials were conducted with different doses and processes to determine the immunizing dose for the clinical trail in Phase III.

In February 2012, designated by the Institute of Medical Biology, Chinese Academy of Medical Sciences, Center for Disease Control and Prevention of Guangxi Zhuang Autonomous Region led the implementation of a multicenter, randomized, double-blind, placebo-controlled clinical trial in Phase III to assess the immune efficiency, safety and immunogenicity of the vaccine in Xing'an and outer counties in Guilin City, Guangxi according to the requirements of *Administration Measures for Drug Registration*, *Good Clinical Practice (GCP)* and *Technical Guidelines for Vaccine Clinical Trials* issued by China Food and Drug Administration. The subjects were 12,000 healthy infants and children 6-71 months old, and the vaccinated participants were randomized in a 1:1 ratio and received either the vaccine or a placebo per 0,28-day immunization procedure. Specifically, 1,100 participants are sorted as immunogenicity subgroup and the venous blood of them are drawn respectively 28d before vaccination and 180d after the whole vaccination to conduct EV71 antibody test. Clinical studies show that the vaccine efficacy of tested vaccine against HFMD caused by Enterovirus 71 is 97.3% (95% CI: 92.6%, 99.0%), and the vaccine efficacy against the serious HFMD caused by enterovirus 71 is 100%, meeting the efficacy target designed in the Proposal. Among experimental vaccine group, the seroconversion rate of susceptible participants reaches to 100% and GMT to 224.37 28d after the whole immunization; among placebo group, the seroconversion rate of susceptible participants reaches to 4.31% and GMT to 7.44. The seroconversion rate and GMT of the former is significantly higher than that of the latter. The seroconversion rate and GMT of experimental vaccine groups remain at 95.81% and 1:118.0 180d after the full immunization. The vaccine is favorable in safety and immunogenicity.

In order to observe the long-term immunogenicity after vaccination, this follow-up research is to assess the antibody level of the immunogenicity subgroup within 1 to 2 years after immunization in the Phase III EV71 clinic research.

2. Research Site and Partners

2.1 Research Site

Yongfu County, Lingchuan County, Lipu County and Xing'an County, from which the participants are selected to set up the immunogenicity subgroup in the original Phase-III clinical research of EV71 inactivated vaccine, are selected as the site for this clinical research.

2.2 Partners

The test for neutralizing antibody in EV71 virus serum is conducted by the National Institutes for Food and Drug Control.

3. Research Purpose and Endpoint

3.1 Research Purpose

Primary Purpose:

To assess the serum antibody titer of EV71 vaccine induced neutralizing antibody in 12th, 18th and 24th months after the participants are vaccinated with two doses of vaccine.

Secondary Purpose:

To assess the seroprevalence of EV71 vaccine induced neutralizing antibody in 12th, 18th and 24th months after the participants are vaccinated with two doses of vaccine.

3.2 Research Endpoint

To research the antibody titer (GMT) and seroprevalence of EV71 neutralizing antibody in the blood specimen.

4. Research Design

To recruit the members of immunogenicity subgroup who participate in Phase-III clinical research and observation of EV71 inactivated vaccine (human diploid cell) conducted by the Institute of Medical Biology, Chinese Academy of Medical Sciences. It needs to complete 2-3ml blood sampling 6 months before and 12 months after enrollment of the participants. Vaccination is excluded from this research.

4.1 Approximate Time of Researchers under Research

The time of participant involved in the research is about three days.

4.2 Research Period

The research began in the last ten-day period of March 2013. The research is completed when all serological data are acquired (determined by the database), expected to be in June 2014.

4.3 Expected Number of Participants

All members of the immunogenicity subgroup in original EV71 Phase-III clinic research are invited to participate in this research.

5. Selection of Participants

The guardian agrees the candidate to accept the product trial after being informed.

Requirements for candidate selection:

■ All members of the immunogenicity subgroup in the safety and immunogenicity observation in original EV71 Phase-III clinic research;

■ The guardians understand the contents and requirements of this research and

voluntarily participate in this research and sign an informed consent form;

■ According to the requirements of this proposal, the participants can accept blood sampling for EV71 neutralizing antibody test.

5.1 Requirements for Candidate Exclusion

The participants cannot participate under the following conditions:

■ The participants are vaccinated with EV71 vaccine that has been registered or is under research after the completion of the original EV71 Phase-III clinic research;

■ The participants suffer from EV71-infected HFMD in the original EV71 Phase-III clinic research;

■ They participants are participating in other researches in the recruitment;

■ The participants have serious acute or chronic illness, abnormal mental conditions or other abnormalities in laboratory examination according to the judgment of the researchers, leading to a significant increase in the risks associated with the research or interpretation to the research results, so such participants are unsuitable to participate in this research.

5.2 Requirements for Withdrawing and Suspension

The clinical observation should be terminated in advance under any one of the following conditions:

■ The guardian requests to withdraw from the clinical observation;

■ The participants are unsuitable to participate in the clinical observation due to poor health;

■ The participants have been vaccinated with EV71 vaccine;

■ The participants stricken by EV71-infected HFMD that is confirmed in the laboratory;

■ The participants have abnormal clinical manifestations and the researchers determine whether the clinical observation for them is terminated in advance.

6. Treatment Research

6.1 Vaccine Research

Vaccination is excluded from this research.

6.2 Allowable Treatment in Research

Allowable vaccine: the participants should not receive the clinical research of other vaccines or other vaccines under research during this clinical research. However, the participant can be vaccinated with routine and emergency vaccines, such as rabies vaccine or tetanus vaccine.

Allowable in the research, if adverse events occurred on participants, they should take necessary medical treatment.

7. Research Steps

See the research process table for specific steps.

Table 1 Research Process

Items	Visit Time		
	Visit upon recruitment	6th month	12th month
Informed consent	×		
Review of enrollment/exclusion requirements	×		
Blood sampling	×	×	×

Note: Blood sampling window period is set as one month.

7.1 Visit upon Recruitment

7.1.1 The researchers explain the *Informed Consent Form of Antibody Immunity Duration Research after Immunization with EV71 Inactivated Vaccine* to obtain the informed consent of the guardian of the volunteers, and then sign the *Informed Consent Form* with the guardians. The form is in duplicate, each party keeping one.

7.1.2 The researchers judge whether the volunteers are qualified to be enrolled and drawn blood.

7.1.3 The researchers draw 2-3ml blood from each volunteer and fill out *Sample Collection Record*.

7.2 Follow-up Visit in 6th and 12th Months

The researchers draw blood 2-3ml each time after judging the participants can withstand blood drawing, and fill out *Sample Collection Record*.

8. Blood Sampling

8.1 Requirements for Blood Sampling

The collected 2-3ml venous blood should be put in the non-anticoagulant tube to conduct serum separation and avoid hemolysis. At least 0.5ml is put in one tube and then submitted for inspection, and the rest serum is put into the sample tube. Serum tube should be pasted with coded label and stored at -20 ℃. If the serum specimens cannot be quickly frozen, firstly refrigerate them within 24h. The serum specimens are sent to the National Institutes for Food and Drug Control for EV71 virus neutralizing antibody test.

All operations from blood collection to serum separation should be aseptic. The serum specimens to be submitted should be frozen at below -20℃, and the serum specimens should be kept low 4℃ in transportation.

8.2 Blood Specimen Coding

First sampling: original research -1

Second sampling: original research -2

Third sampling: original research -3

For example: As for the participants numbered as 0600001, the blood specimens of three times are coded as 060001-1, 060001-2 and 060001-3 respectively.

9. Data Analysis/Statistical Methods

See Statistical Analysis Plan (SAP) and/or clinical research report for specific analysis items, including missing data, and unused and forged data if applicable. The deviations in the statistical analysis plan should be recorded in the clinical research report.

10. Quality Assurance and Monitoring for Clinical Research

10.1 Training for Researchers

The researchers refer to all staff involved in this research should possess medical care professional qualifications (including practitioner qualification certificate, medical and technical certificate), and should undertake this research only after qualified in the following training.

- Clinical observation scheme;

- Informed consent form;

- Blood specimen collection record and other relevant forms;

- Venous blood collection and serum separation technology;

- *Good Clinical Practice* (GCP)

10.2 Supervisor

10.2.1 Requirements

According to Article 35 of Administration Measures for Drug Registration, the Sponsor shall designate a professional to supervise the implementation of *Good Clinical Practice*. Field supervisor shall undertake supervision through the research and make sure that the clinical research is performed in accordance with GCP and clinical research plan and completed in a timely manner. The supervisor shall be acquainted with the following items:

- *Good Clinical Practice* (GCP) and various laws and regulations;
- Clinical research plan;
- Informed consent form;
- Blood specimen collection record and other relevant forms;
- Venous blood collection and serum separation technology;
- Site data acquisition

10.2.2 Responsibilities

- Prior to clinic research, the Supervisor shall confirm whether the research institute is qualified from the following aspects: whether the staffing and training are sufficient, laboratory facilities are complete and in favorable running state, the institute is capable to conduct various tests related to this research, or the involved researchers are familiar with the requirements of the clinic research plan.

- During the research, the Supervisor should supervise the implementation of the clinic research plan by the researchers, make sure that all informed consent forms of the participants are acquired prior to the research, and ensure all participants are suitable for the research.

The Supervisor should make sure all related records and reports of the data are correct and complete and all forms are filled out correctly, and ensure that all

mistakes or omissions are rectified or indicated with signature and dating of the researcher.

■ The supervisor should also verify that blood specimens are collected, separated, preserved and transported in accordance with the plan, and take down relevant records.

■ The Supervisor should assist the researchers in notification and application issues and inform the Sponsor of the data and results of clinic research.

■ The Supervisor should regularly supervise the research on site and submit written supervision report to the Sponsor and the chief researcher of the research institute in a timely manner (within 3 days). The report should include supervision date, specific time, supervisor name, and supervision results, as well as any follow-up visits, clinic researches and inspected the researchers fail to perform, and whether the mistakes and omission are rectified.

■ The Supervisor should resolve any deviation in clinical research jointly with the researchers, and inform the Ethics Committee and the pharmaceutical supervisory and administrative departments of major events.

10.3 Safety of Participants

Vaccination is excluded from this research. Blood sampling should be conducted by qualified nurses in township hospitals or the clinics of disease control centers. The syringes, lancets and tubes for blood drawing should be made of disposable sterile materials to avoid cross-infection. The researchers should assess whether the participants are suitable for blood sampling. In blood drawing, the participants may cry in pain and the drawing part may bruise, but researchers should try to avoid above issues. Meanwhile, the researchers should pay close attention to the conditions of the participants in blood drawing, and the doctor will conduct timely and proper treatment against uncomfortable symptoms. If serious adverse reactions are caused by this research, the research institute should contact the Sponsor to pay associated medical expenses and provide some compensation according to the

severity of adverse reactions.

10.4 Specimen Management

Serum separation should be conducted on the same day the non-anticoagulant blood specimens are taken from the participants to prevent against severe hemolysis. Blood specimens should be preserved at -20℃ (which should be monitored every day) and transported in a refrigerated way to the appointed trial laboratory after all field works are completed.

10.5 Instrument Calibration and Thermometer & Glassware Standardization

- Thermostat incubator is within the validity period, attached with temperature records, and has calibrated.
- The refrigerator is within the validity period, attached with temperature records, and has calibrated.
- Thermometer and other measuring tools has been standardized.
- The syringes for blood sampling should be disposable, attached with batch number and validity mark, and manufactured by such factories that have national production license.

10.6 Clinical Research Data Management

According to Article 48 and Article 49 in Chapter 8 of Good Clinical Practice, records, reports and other original data of the clinical research should be kept intact. All operations in the clinic research should be recorded in relevant forms in a timely, accurate, complete, normative and authentic manner. The forms should not be modified unless necessary. In case of modification, the form should be kept clean and recognizable, and signed and dated by the modifier.

To ensure that statistical data of clinical research is true and reliable, the researchers

should review all on-site records, and the supervisors should conduct scheduled and unscheduled audits for the data records until the form is completed, and carefully verify, review and audit the contents of the forms before collecting them. Audit results should be recorded, and blood specimen delivery documented.

All information should be added up and processed by the professional statisticians of the agency or department that is entrusted by the clinical research institute or the Sponsor.

For severe and Level-3 adverse events, specific details should be described in the case form and serious adverse event record.

10.7 Supply and Preservation of Research Data

- Serum testing results (testing institute, the Sponsor and research institute)
- Clinical research (the Sponsor and research institute)
- Authorization letter to and acceptance letter of the clinical research institute (the research institute and the Sponsor)
- Approvals from the Ethics Committee (the original kept by the research instate and the copies kept by the Sponsor)
- Investigation and reports of adverse events (SFDA, the Ethics Committee, the research institute and the Sponsor)
- Other field original data (signed informed consent form and blood collection registration form): kept by the on-site disease control and prevention center for five years since the completion of the clinic research.

Other data:

Blood specimen submission and delivery records should be kept by the research institute, while serum testing records and results kept by the laboratory.

10.8 Agreement and Clinical Research Report

- Original of the Agreement: in triplicate; 3 kept by the Sponsor and 1 by the research institute.

- Clinical summary report (stamped with red seal): in quintuplicate; 1 archived by Center for Disease Control and Prevention of Guangxi Zhuang Autonomous Region and 4 kept by the Sponsor.

11. Timetable

Gross research duration: 15 months

12. Ethics Committee

12.1 Auditing and Approving Documents for Clinical Research

- Clinical research plan;

- Informed consent form;

- Resumes of chief researchers

12.2 Supervision over Implementation

12.2.1 Informed Consent

The Ethics Committee should work out the selection orientation for participants, check whether the information provided to the participants/guardians/legal representative is complete and comprehensible, and whether the approach to get informed consent is suitable. Throughout the clinic research, the Ethics Committee should supervise whether there is any ethic problem which may cause harm to the participants and whether the participants who suffer from the clinic research are treated or compensated or secured with relevant insurance measures, and assess the risks taking by the participants.

12.2.2 Confidentiality

The Ethic Committee should make sure that the personal information of all participants is confidential through the process from clinic research, biological sampling, reporting to publishing. Biological specimen should be marked only with ID number or laboratory number. It is strictly required that only the chief researchers are entitled to get electric or written copies.

12.2.3 Potential Dangers and Minimization Thereof

The Ethic Committee should, 1) if any adverse reaction is identified to be caused by blood drawing, check whether the participants are treated promptly as required; 2) in case of any life-threatening event, whether the participants are sent to the hospital immediately for treatment and whether the measures taken are reported. 3) whether associated measures are taken to make sure that the venous blood is sample by experienced and trained medical personnel according to the procedures under strict supervision to minimize the pains to the participants as well as local infection caused by venipuncture.

13. Adverse Event Treatment

13.1 Definition

Adverse Event refers to the unexpected medical events that are happened on the participant in clinical research and may not be in a causal connection with the vaccine or vaccination.

Adverse Reaction refers to the unexpected or harmful reactions that happen in the vaccination which is conducted with specified dosage as per the procedures, normally related to vaccination.

Serious Adverse Reaction refers to life-threatening events such as death,

hospitalization, prolongation of hospitalization, persistent disability, failure of self-care ability, and other events related to clinical research, specifically including epilepsy, coma, tetany, diabetic ketoacidosis, disseminated intravascular coagulation, disseminated ecchymos, numbness or paralysis, acute mental disorder, and severe depression.

13.2 Notification and Treatment

The researchers should make the participants understand that they should report any diseases and abnormal events occurring after vaccination and acquired with the appropriate notifying approaches.

According to relevant nation provisions, the participants should be properly treated at the designated hospital. The researchers should conduct investigation and follow-up visits for the adverse reactions, including illness history review, physical examination, necessary laboratory tests and treatment, and tracking until the events are solved, while filling out the detailed investigation form and keep follow-up records. Questionnaire includes symptoms, signs, diagnosis and laboratory results.

The researcher should inform the pharmaceutical supervisory and administrative department, the Sponsor and the Ethics Committee of any serious adverse events within 24h since it occurrence, and the Sponsor should notify the pharmaceutical supervisory and administrative department and other researchers involved in the clinic research of the same drug in accordance with the regulations.

14. Appendixes

1) The approvals from the Ethic Committee;

2) Sample of informed consent form;

3) Information & qualifications of clinical research institute, and resumes & authorization table of the chief researchers;

4) Clinical research plan and modifications thereof, and the approvals of the Ethic Committee for the modifications;

5) Case report of serious adverse events and adverse events regarded as critical by chief researchers;

6) Statistical analysis report;

7) Clinical research report (serum testing report)

15. References

1) *Technical Guidelines for Clinical Vaccine Trials* (GSYJZ [2004] No. 575) issued by SFDA on December 3, 2004

2) *Good Clinical Practice* (GCP) issued by SFDA in June 2003

3) Combination of Vaccine Quality Control and Clinical Research Technical Guide (GSYJZ [2005] No. 493): Part 5, issued by SFDA on October 14, 2005

2013年 3月 20日

1.研究概况

由中国医学科学院医学生物学研究所研制的 EV71 灭活疫苗（人二倍体细胞）用于预防肠道病毒 71 型（EV71）的感染，特别是预防重症手足口病的发生。2012 年，该疫苗在广西壮族自治区桂林市兴安等七县开展了一项多中心、随机、双盲、安慰剂对照的Ⅲ期临床研究，研究对象为 12000 名 6-71 月龄健康婴幼儿及儿童，其中有约 10%的对象为免疫原性亚组。临床研究结果表明，该疫苗具有良好的安全性和免疫原性。为了进一步观察该疫苗接种后的长期免疫原性，本研究拟对原Ⅲ期临床研究中的免疫原性亚组对象的疫苗免疫后 1-2 年的抗体水平进行评价。

2.研究程序

本次研究的对象为原Ⅲ期研究中完成全程观察的免疫原性亚组受种者。为保证结果的真实、可靠，参加本研究时您及您宝宝将需要配合医生完成以下工作：

1) 在理解本研究的过程和要求后，同意参加研究并完全自愿签署知情同意书；
2) 研究开始前医生会询问您宝宝的近期病史和健康状况，如符合要求，医生会将您宝宝编入组并使用原研究编号以识别身份，您宝宝的信息将会被严格保密；
3) 本研究无需接种任何疫苗，您的宝宝只需要采集 3 次血样，每次 2-3 毫升（约手指肚大小量），进行抗体检测以了解接种效果。采集时间为完成全程两剂疫苗接种后的 1 年、1 年半和 2 年（医生会通知您）。

3.风险和获益

采血过程中可能您宝宝会因疼痛而哭闹，还可能出现采血部位的淤肿等（我们会选择有经验的护士进行操作以尽量避免）。同时医生会密切关注您宝宝，当出现不适症状时医生会进行及时、良好的治疗。如因本研究引起的严重不良反应，我们会负责支付相应的医疗费用，并根据不良反应的严重程度提供一定的补偿。

参加本次研究的所有体检和抗体检查都是免费的，通过采血可以了解您宝宝体内的 EV71 抗体变化情况。研究期间医生会一直对您宝宝的健康状况进行密切关注。由于您参与了本次临床研究，将有助于疫苗研究和对疾病预防控制做出贡献。

同意 永洪 谢 2013年 3月 20日

4.经济补偿

我们将对您为本研究所付出的时间、交通和误工做出适当的经济补偿。

5.保密约定

涉及您及宝宝的所有个人信息和检测结果会被严格保密。研究结果可能刊登于科学杂志，但不会列明您/您孩子的任何身份资料。所采集的血清样本仅用于本次研究。同时，您不能将本次研究的有关信息向其它的厂家或研究机构透露。

6.中途退出

我们十分希望您宝宝全程参加本研究，但您可以选择继续参加或退出，且中途退出您宝宝的权益不会受到任何影响。研究中出现任何问题可随时与医生联系。

联系电话：**广西区疾病控制中心（南宁）　　莫兆军医师（15177771508）**

关于您作为参加者的权益问题，请联系：

广西伦理审查委员会（南宁）　　谢永洪　　0771-2518979

7.签署知情同意书

我已详细阅读并理解了以上全部内容，医生也已向我作了详细的解释；我已理解和认可该研究的所有要求，我及我宝宝自愿参加该临床研究，并同意在知情同意书上签署姓名。

宝宝姓名：__________________

宝宝监护人（签名）：_______________关系：____________ 日期：____________

我保证已向受种者监护人（其父亲、母亲）介绍了本研究的相关情况并进行了充分的交流和沟通，在监护人协助下我获得了以上信息：

医生 （签名）：_________________________ 日期：________________________

INFORMED CONSENT FORM FOR RESEARCH ON ANTIBODY PERSISTENCE OF EV71 INACTIVATED VACCINE

1. Research Overview

EV71 inactivated vaccine (human diploid cell) developed by the Institute of Medical Biology, Chinese Academy of Medical Sciences is intended for preventing enterovirus 71 (EV71) infection, especially hand-foot-mouth disease. In 2012, a multi-center, randomized, double-blind, and placebo-controlled phase III clinical research of EV71 on 12,000 healthy infants and children aged between 6 to 71 months in Xing'an County and other 6 counties of Guilin City, Guangxi Zhuang Autonomous Region; about 10% research participants fall into immunogenicity subgroup. Research results indicate that EV71 is of good safety and immunogenicity. This research is intended to assess the antibody level of those research participants under the immunogenicity subgroup of previous phase III clinical research during 1~2 years after vaccination and thus to observe the long-term immunogenicity of vaccine.

2. Research Procedures

This research is carried out on those vaccinated participants under the immunogenicity subgroup throughout previous phase III clinical research. To achieve real and reliable results, you and your child shall assist the doctor in the following aspects:

1) You are required to sign the *Informed Consent Form* out of your own accord after you fully understand and agree to all research procedures and requirements.
2) Doctors will inquire you of your child's recent medical history and physical conditions before research. If all relevant requirements are met, your child will be arranged into corresponding group and identified via previous research code. All information about the child will be kept under wraps.
3) This research involves no vaccination but 3 blood samples for each child. 2~3ml blood (nearly equivalent to volume of the finger pad) will be sampled each time

for antibody detection and vaccination inspection. Sampling will be conducted at 1, 1 and half, and 2 year/years later after 2 doses of vaccine are injected (You will receive notice from doctors).

3. Risks and Benefits

Your child may cry from pain or get bruised at sampling parts (trained nurses will be appointed for sampling to avoid above cases as much as possible). Close attention will be paid to the child; any discomfort will be immediately and properly treated by doctors. In case of serious adverse reaction due to this research, all corresponding medical expenses and compensation based upon degree of severity will be born by our institute.

All physical examinations and antibody inspections relating to this research are free. Specific information about EV71 antibody in child's body can be obtained via blood sampling. Close attention from doctors will be paid to the child's physical conditions during research. You and your child's participations contribute to vaccine study and disease prevention.

4. Compensations

Proper compensations will be provided to you for time, transportation fee and delay in work.

5. Confidentiality

All individual information and detection results involving you and your child will be confidentially treated. Research results may be published in science magazines but none of you and your child's identity data will be indicated. All serum samples will be only applied in this research. You are required not to disclose information related to this research to any other plant or research institute.

6. Dropout

You and your child are badly wanted for this research, but you are allowed to

continue or drop out. Dropout will exert little impact on your child's rights. In case of any problem during the research, doctors are at your service anytime.

Contact:

Center for Disease Control and Prevention of Guangxi Zhuang Autonomous Region (Nanning)

Doctor Mo Zhaojun (15177771508)

Contact for information about related rights:

Guangxi Institutional Review Board (Nanning)

Xie Yonghong (0771-2518979)

7. Signing *Informed Consent Form*

I have read carefully and understood all above contents and also received specific related descriptions from doctors. I understand and agree to all relevant requirements; I and my child are willing to take part in this research and sign the *Informed Consent Form*.

Child's Name: _____________

Child's Guardian (Signature): __________Relation: _______Date: ________

I guarantee that the guardian of research participant (his/her father or mother) receives my descriptions about this research and full communications between us are accomplished. I acquire the above information with the assistance from the guardian.

Doctor (Signature): _______________ Date: ________________

Version No.: 1.0 Version Date: March 18, 2013

Table S1 Baseline characteristics of the participants in the analysis *

Characteristic	Vaccine group (N=549)				Placebo group (N=550)			
	6-to-11 months	12-to-23 months	24-to-35 months	36-to-71 months	6-to-11 months	12-to-23 months	24-to-35 months	36-to-71 months
Age--months								
Mean	9.25±1.77	17.50±3.28	29.28±3.39	51.16±9.99	9.34±1.78	17.86±3.45	29.44±3.37	50.93±10.19
Sex--number(%)								
Male	85(48.57)	94(54.02)	76(50.67)	24(48.00)	88(50.29)	107(61.14)	79(52.67)	22(44.00)
Female	90(51.43)	80(45.98)	74(49.33)	26(52.00)	87(49.71)	68(38.86)	71(47.33)	28(56.00)

* Plus-minus values are means ± SD. There were no significant differences between vaccine group and placebo group at baseline.

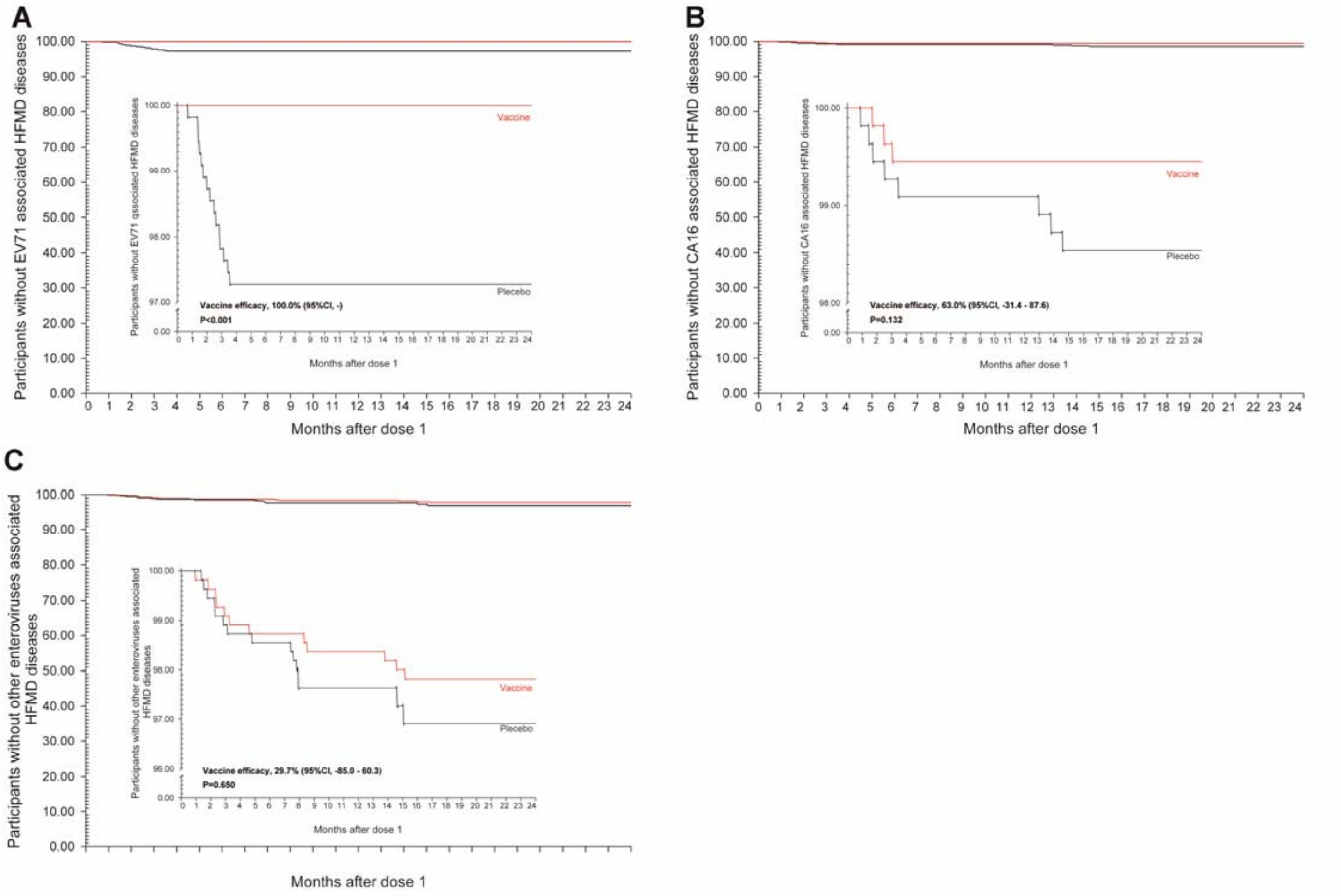

Figure S1. Cumulative cases of HFMD reported in 1,100 participants

A total of 1,100 participants, receiving either the vaccine or placebo, were observed during the surveillance period. The cumulative curves for the cases of HFMD induced by enterovirus 71 (EV71), coxsackievirus A16 (CA16), and other enteroviruses were estimated as a percentage among these participants with Kaplan–Meier survival curves during the period from the receipt of the first dose until 24 months thereafter. The inset shows the same data on an enlarged y-axis. Differences in the distributions of cases of HFMD among the individuals who received the placebo and those who received the vaccine were evaluated with a log-rank test. (A) Cumulative curve for the cases of HFMD induced by EV71. (B) Cumulative curve for the cases of HFMD induced by CA16. (C) Cumulative curve for the cases of HFMD induced by other enteroviruses.
